# Dietary N-Carbamylglutamate Modulates Muscle Growth, Nutrient Composition, and Metabolic Profiles in Danzhou Chickens

**DOI:** 10.3390/foods15193516

**Published:** 2026-10-01

**Authors:** Dexin Zhao, Haoliang Chai, Xilong Yu, Fengjie Ji, Weiqi Peng, Hongzhi Wu

**Affiliations:** 1 Tropical Crops Genetic Resources Institute, Chinese Academy of Tropical Agricultural Sciences, Haikou 571101, China; 2 College of Animal Science and Technology, Northeast Agricultural University, Harbin 150030, China; 3 College of Animal Science and Technology, Northwest A&F University, Yangling, Xianyang 712100, China

**Keywords:** N-carbamylglutamate, Danzhou chicken, muscle growth, nutrient composition, metabolic profiles

## Abstract

This study evaluated dose-dependent effects of dietary N-carbamylglutamate (NCG) on muscle growth, nutrient composition, and metabolic profiles in Danzhou chickens. A total of 480 one-day-old female Danzhou chicks were randomly assigned to diets supplemented with 0, 400, 800, or 1200 mg/kg NCG for 35 days and slaughtered at 35 days of age. NCG at 400–800 mg/kg improved growth performance, increased crude protein and free amino acids (arginine, methionine), and altered fatty acid composition in a muscle-specific manner, with increased breast-muscle MUFA and decreased leg-muscle arachidonic acid and DHA; total PUFA did not differ. Histological analysis showed that NCG increased leg-muscle fiber cross-sectional area and Feret diameter, accompanied by a reduction in fiber density. Mechanistically, NCG altered the mRNA expression of *IRS2, IGF1*, *MyoG*, *AKT1*, and *FOXO1* in a manner consistent with modulation of the IGF-1/IRS2/PI3K/Akt/FoxO1 axis. Metabolomics suggested an association with purine metabolism; however, this finding is exploratory and does not establish causality. Integrated multi-omics revealed crosstalk between purine and lipid metabolism. Among the doses tested, 800 mg/kg produced the largest response for several endpoints; however, no formal dose-optimization model was fitted, and the optimal dose remains to be determined. The 1200 mg/kg dose did not further improve most endpoints, indicating a nonlinear dose response. This study provides the first multi-omics evidence demonstrates that NCG modulates muscle growth, nutrient composition, and metabolic profiles in indigenous chickens through coordinated regulation of anabolic signaling, purine metabolism, and lipid remodeling. Direct meat-quality measurements were not performed; therefore, effects on eating quality, technological quality, oxidative stability, and consumer preference remain to be established.

## 1. Introduction

Chicken is a globally dominant high-quality protein source, with yield, nutrition and edible traits governing poultry industrial profits and market competitiveness [1,2]. As a core staple food, chicken meat has long been a focal food science research topic for its balanced nutrition and sustainable industrial value [3,4]. Danzhou chicken, a Hainan indigenous breed listed in China’s National Livestock and Poultry Genetic Resources Catalogue in 2023, carries unique genetic and breeding value and lays a foundation for related industrial research [5]. It has gained popularity among consumers for its rich flavor, thin bones and compact muscle texture, and is suitable for cooking methods such as poaching and salt baking, with promising prospects in high-end poultry markets [6]. However, large scale breeding of this breed faces multiple bottlenecks including uneven individual growth, inadequate muscle tenderness and room for nutritional optimization. Most importantly, molecular mechanisms that regulate its muscle growth remain unclear, which limits targeted breeding for superior meat quality.

Given that myofiber morphology dictates meat tenderness, and amino/fatty acid profiles determine flavor and oxidative stability, the regulatory pathways governing muscle protein metabolism are directly relevant to meat quality traits. Given that myofiber morphology is associated with meat tenderness, and amino/fatty acid profiles may influence flavor and oxidative stability, the regulatory pathways governing muscle protein metabolism are relevant to meat quality traits. Muscle growth relies on the dynamic balance between protein synthesis and degradation, and the efficiency of this balance influences carcass yield, myofiber morphology, and the sensory and nutritional attributes of meat—including tenderness (which may be influenced by myofiber size and density), flavor (which may be associated with amino acid and nucleotide profiles), and oxidative stability (which may be influenced by fatty acid composition) [7,8,9,10,11]. The *insulin-like growth factor 1* (*IGF1*)/*insulin receptor substrate 2* (*IRS2*)/*protein kinase B* (*AKT1*)/*forkhead box O1* (*FOXO1*) signaling cascade serves as a master regulatory hub that orchestrates poultry muscle protein turnover [12,13]. In essence, upon activation by upstream cytokines, this axis concurrently promotes anabolic signaling via the mTOR pathway and suppresses catabolic gene transcription (e.g., *Atrogin-1* and *MuRF1*) through *AKT1*-mediated *FOXO1* phosphorylation and cytoplasmic sequestration [14,15,16,17,18]. However, direct evidence for IRS2/PI3K/Akt/FoxO1 activation in chicken skeletal muscle is still limited, and the present study therefore examined mRNA expression of key genes in this pathway as an exploratory analysis. The net outcome of this pathway is the regulation of myofiber proliferation, differentiation, and hypertrophy, which directly shapes the structural framework of muscle tissue [19]. Given that myofiber morphological traits, alongside the accumulation of free amino acids and the remodeling of fatty acid profiles, constitute the core determinants of chicken meat’s nutritional value, flavor, and oxidative shelf-life, understanding the regulatory logic of the IGF1/AKT1/FOXO1 axis is of paramount importance for improving the overall quality of indigenous poultry meat [20,21]. Notably, for Danzhou chickens, whose market competitiveness relies heavily on their superior intrinsic texture and flavor, elucidating how this pathway responds to nutritional interventions is a prerequisite for developing breed-specific strategies that enhance both growth efficiency and premium meat traits, rather than focusing solely on production yield.

*N*-carbamylglutamate (NCG) shares structural similarity with *N*-acetylglutamate and functions as a key intermediate of the urea cycle. It can activate *carbamoyl phosphate synthetase I* (*CPS-I*), the rate-limiting enzyme for endogenous arginine synthesis in livestock and poultry [22,23]. NCG facilitates glutamine and proline conversion into arginine inside organisms without extra arginine supplementation [24,25]. NCG has been shown to increase circulating arginine and improve muscle growth in broiler chickens [26]. NCG has been studied as a means to enhance endogenous arginine synthesis in animals. However, arginine metabolism in birds differs from that in mammals, and direct evidence for the CPS-I–arginine pathway in poultry remains limited. Therefore, the rationale for using NCG in chickens is based on its proposed role in enhancing arginine availability, rather than on a fully established poultry-specific urea-cycle mechanism.

Arginine acts as a precursor for nitric oxide and polyamine synthesis, and participates in protein metabolism, immune regulation and hormone secretion [27,28]. As a novel functional feed additive, NCG improves animal metabolic balance and production performance [24,25,29,30,31,32]. In Danzhou chickens, our previous study showed that NCG promotes growth and immunity via gut microbiota–metabolite interactions involving sphingolipid and mTOR pathways [33]. However, its effects on muscle growth, nutrient composition, and metabolic profiles remain unclear. Existing research rarely explores the effects of NCG on Danzhou chicken muscle development and the underlying regulatory mechanisms. Most relevant studies test only individual phenotypic traits or pathway genes and lack multidimensional research systems integrating growth performance, muscle morphology, amino acid and fatty acid nutrition, and multi-omics molecular analysis. Such research gaps hinder comprehensive clarification of molecular networks through which NCG modulates chicken muscle growth.

In this study, Danzhou chickens were selected as experimental subjects. NCG-supplemented diets were used to assess growth performance, muscle morphological characteristics, and muscle amino acid and fatty acid composition. Together with muscle transcriptome and metabolome data, these measurements clarify the regulatory effects of NCG on Danzhou chicken muscle growth and meat quality. It further reveals how NCG promotes muscle protein synthesis through the IGF1/IRS2/AKT1/FOXO1 pathway. The outcomes provide scientific evidence for rational application of NCG in native high-quality chicken breeding, and offer theoretical support for quality improvement and industrial development of Danzhou chicken.

## 2. Materials and Methods

### 2.1. Experimental Materials and Animals

NCG with a purity exceeding 99.5% was sourced from Sigma-Aldrich, St. Louis, MO, USA. The NCG concentrations in the experimental diets were analyzed by HPLC/LC-MS and were 398.2, 801.5, and 1196.8 mg/kg for the N1, N2, and N3 diets, respectively, which were close to the target concentrations. One-day-old female Danzhou chicks with a consistent genetic background and healthy physical condition were supplied by Hainan Rekeyuan Ecological Breeding Co., Ltd., Danzhou, China. Female chicks were identified at hatch by vent sexing performed by trained hatchery personnel, and only chicks confirmed as female were included. All animal trials took place within standardized poultry housing at the Tropical Crops Genetic Resources Institute, Chinese Academy of Tropical Agricultural Sciences.

### 2.2. Experimental Design and Animal Care and Use

Unless otherwise stated, the animal management, slaughter, sampling, histological, transcriptomic, and metabolomics procedures were performed as described in our previous study [33], with minor modifications. A total of 480 one-day-old female Danzhou chicks were used in this study. A single factor fully randomized trial design layout was adopted for this research. All experimental chicks were derived from a single hatchery batch (same batch of hatching eggs, same hatch date). Each pen measured 1.2 m × 1.2 m (1.44 m^2^) and housed 20 birds, corresponding to a stocking density of 13.9 birds/m^2^ (0.072 m^2^/bird). Before randomization, pens were blocked by house location. Within each block, pens were randomly assigned to the four dietary treatments using a Microsoft Excel random number generator. The pen was the experimental unit for growth performance (*n* = 6 pens per treatment). The control group received an unsupplemented basal diet, while the three experimental groups were fed basal diets supplemented with 400, 800 and 1200 mg/kg NCG, designated N1, N2 and N3, respectively. A preliminary trial showed that 400–800 mg/kg NCG was effective, and 1200 mg/kg was selected as a high-dose challenge level. For growth performance, the pen was the experimental unit (*n* = 6 pens per treatment). Body weight was recorded individually for all birds at day 1 and day 35. Individual body weights were then averaged within each pen to generate pen-level values for statistical analysis. For tissue-related measurements, two birds per pen were used (*n* = 6 birds per treatment). Feed conversion ratio (FCR) was calculated on a pen basis (*n* = 6 pens per treatment) as: FCR = total feed intake per pen (kg)/total body weight gain per pen (kg). Total feed intake per pen was recorded as feed offered minus feed remaining. Total body weight gain per pen was calculated as the final total body weight of surviving birds minus the initial total body weight of all birds placed in the pen. Mortality was recorded daily. Because individual feed intake of dead birds was not measured, no correction for dead-bird feed intake was applied; feed consumed by dead birds remained included in the pen-level total feed intake. Given the very low mortality rate (only 2/120 birds in the N3 group, 1.7%), this is unlikely to have materially affected FCR or the between-group comparisons. A sensitivity analysis assuming dead birds consumed the pen-average feed intake did not change the conclusions. Formulation of the basal feed followed the GB/T 5916-2020 formula feeds for layers and broilers standard [34], with full composition laid out in Table S1. The nutrient composition of the experimental diets was determined according to the following Chinese national standard methods: dry matter (DM) by GB/T 6435-2014 [35], crude protein (CP) by GB/T 6432-2018 [36], ash by GB/T 6438-2025 [37], calcium (Ca) by GB/T 6436-2018 [38], phosphorus (P) by GB/T 6437-2018 [39], neutral detergent fiber (NDF) by GB/T 20806-2022 [40], and acid detergent fiber (ADF) by GB/T 20805-2022 [41]. Organic matter (OM) was calculated as DM minus crude ash. The calculated nutrient levels of the diet were obtained from the Feed Database in China Feed Database (2020). All feed batches underwent uniform blending before distribution. The target NCG concentrations were 400, 800, and 1200 mg/kg. This is a limitation of the present study, and possible losses during mixing, storage, or processing cannot be excluded.

Light management matched the developmental rhythm of young chicks. Birds aged one to three days received 23 to 24 h of daily light with a one-hour dark interval to buffer unexpected power cuts. Daily light duration was reduced to 20 to 22 h for four- to seven-day-old chicks, then gradually reduced to 16 h by week two or three, and further adjusted to 14 to 15 h at four to five weeks of age. Light intensity shifted accordingly, held at 30 to 40 lux in the first week and lowered to 10 to 20 lux from week two onwards, with LED lamps deployed to eliminate sharp light fluctuations and reduce stress. A standardized vaccination schedule was strictly implemented: Marek’s disease vaccine was administered on the day of hatch, combined Newcastle disease and infectious bronchitis vaccines were given at seven and 21 days, and infectious bursal disease vaccine was injected on day 14. Strict disinfection of injection tools and precise dose control prevented missed or duplicate immunization.

House temperature followed a gradual cooling gradient. A temperature of 34 to 35 °C was maintained for newly hatched chicks and lowered by 2 to 3 °C each week until it stabilized at 24 to 26 °C by four to five weeks, with even heat distribution maintained across the entire housing space. Feeding frequency was adjusted with chick age: six to eight meals daily for one- to two-week-old birds and four to six meals daily from weeks three to five. All treatment groups (CON, N1, N2, and N3) received the same Jiyesheng Electrolyte Multivitamin supplement in the drinking water. The supplement (Jiyesheng Electrolyte Multivitamin, Wuhan Jiyesheng Chemical Co., Ltd., Wuhan, Hubei, China) was provided at 125 g per 100 L of water. Its labeled composition was as follows (per kg): vitamin A, 11,023,000 IU; vitamin D3, 1,653,450 IU; vitamin E, 5512 mg; vitamin K3, 4409 mg; vitamin B1, 551 mg; vitamin B2, 1102 mg; vitamin B12, 20 mg; calcium pantothenate, 8812 mg; folic acid, 276 mg; sodium, 7.5 g; potassium, 10 g. Vitamin B6, niacinamide, biotin, vitamin C, chloride, magnesium, and calcium were not separately declared on the product label of Jiyesheng Electrolyte Multivitamin. The same dosage and administration schedule were used for all groups. Continuous ventilation ran throughout the rearing cycle to minimize environmental stressors and sustain robust growth.

### 2.3. Sample Collection

Slaughter was performed at the experimental facility of the Tropical Crops Genetic Resources Institute, Chinese Academy of Tropical Agricultural Sciences, in a designated slaughter room adjacent to the poultry housing.

At trial termination, all birds underwent a 12 h fasting period with unlimited access to water. Two individuals from each replicate pen were randomly selected for slaughter, following the *AVMA Guidelines for the Euthanasia of Animals: 2020 Edition*. Specifically, birds were electrically stunned (50–70 V, 50 Hz for 5–10 s, Water Stunner 032, BAADER, Hamburg, Germany) and then immediately exsanguinated by severing the jugular vein or carotid artery. These parameters were selected in accordance with the WOAH Terrestrial Animal Health Code and the AVMA Guidelines for the Euthanasia of Animals: 2020 Edition. A low-frequency (50 Hz) sinusoidal AC with a minimum current of 100 mA per chicken and a duration of at least 4 s is recommended for poultry water-bath stunning; the 5–10 s duration used here exceeds this minimum. The voltage was adjusted according to the manufacturer’s instructions to achieve the required current. The equipment was calibrated before use, and stunning effectiveness was verified by the absence of the nictitating membrane reflex. The exact time elapsed from exsanguination to sample collection was not recorded separately for each analysis type. However, all sampling procedures were performed immediately after exsanguination in a fixed order, and all samples were collected and processed within 15 min after exsanguination. Therefore, postmortem changes in pH, proteolysis, water distribution, IMP degradation, free amino acids, texture, and flavor precursors cannot be excluded. Routine meat quality samples were collected first, followed by multi-omics samples, and finally histological samples. Breast and leg muscles were collected from the same bird. Specifically, the right breast muscle and the right leg muscle were used for histological, and omics analyses, while the corresponding left-side muscles were retained as backup samples. During excision, the skin and visible connective and adipose tissues were carefully removed. Samples intended for proximate composition, free amino acid, fatty acid, and IMP analyses were sealed in zip-lock bags and stored at −20 °C for no more than two weeks before analysis. Three additional portions of each breast and leg muscle were separately transferred to 2 mL cryotubes, snap-frozen in liquid nitrogen, and stored in an ultralow-temperature freezer at −80 °C for multi-omics analyses. Muscle strips roughly two to three centimeters long, cut parallel to muscle fibers, were rinsed using 0.9% saline and fixed in 4% paraformaldehyde, preserving tissue integrity for subsequent histological observation.

### 2.4. Growth Performance Assessment

Individual live weight at day 1 and day 35, alongside daily feed consumption on a pen basis, formed the core measurement indicators. At day 1, all chicks were individually weighed before random allocation. At day 35, all surviving birds were individually weighed. Individual body weights were then averaged within each pen to obtain pen-level initial body weight (IBW) and final body weight (FBW). Feed intake was recorded on a pen basis. Average daily gain (ADG), average daily feed intake (ADFI), and feed conversion ratio (FCR) were calculated for the 1–35-day period using the pen as the experimental unit (*n* = 6 pens per treatment). Intermediate live body weights were not recorded because the present trial was designed to evaluate the overall 1–35 d effects of graded NCG supplementation on final growth performance and meat quality, rather than to characterize weekly growth curves or compensatory growth. In addition, a single basal diet was used throughout the 35 d trial, with no starter–grower–finisher dietary transition; therefore, phase-specific ADG, ADFI, or FCR values were not required. Limiting handling to day 1 and day 35 also minimized repeated catching and weighing stress, which could confound feed intake and growth in this indigenous breed. We acknowledge that this design cannot detect compensatory growth or phase-specific growth responses, and this limitation is noted in the Discussion. Future studies requiring growth trajectory data should include weekly or phase-based weighing.

### 2.5. Conventional Nutrient and Inosinic Acid Detection

Muscle moisture content relied on the direct drying method specified under GB 5009.3-2016 [42]. Roughly 2.0000 g muscle tissue was weighed and baked at 101 to 105 °C until constant mass, with moisture content calculated from weight loss. Crude protein quantification adopted the Kjeldahl method per GB 5009.5-2016 [43], involving sulfuric acid digestion, alkaline distillation and boric acid absorption, followed by titration with standard hydrochloric acid and computation using a conversion coefficient of 6.25. Crude fat was extracted through Soxhlet reflux in line with GB 5009.6-2016 [44], using petroleum ether with a boiling range of 30 to 60 °C; fat yield was determined after solvent recovery. Crude ash content was determined by high-temperature incineration as outlined in GB 5009.4-2016 [45], with samples carbonized and heated at 550 ± 25 °C to constant weight; ash content was calculated from residual inorganic mass.

Muscle inosinic acid (IMP) concentrations were quantified by high-performance liquid chromatography (HPLC). Separation utilized a Waters Symmetry C18 column (250 mm × 4.6 mm, 5 μm), with isocratic elution via pH 6.5 ± 0.1, 0.05 mol/L potassium dihydrogen phosphate buffer prefiltered through a 0.45 μm membrane and ultrasonically degassed. The flow rate was 1.0 mL/min with the column temperature fixed at 30 °C, and a diode array detector (DAD) detector captured signals at a wavelength of 248 nm with a 10 μL injection volume. External standard calibration covered IMP concentrations from 0.02 to 0.32 mg/mL, generating standard curves with R^2^ above 0.999. All samples were analyzed in triplicate, requiring relative standard deviation (RSD) values below 5% to validate data stability.

### 2.6. Free Amino Acid Profile

Free amino acids (FAAs) were detected with an amino acid analyzer (L-8900, Hitachi, Tokyo, Japan). One gram of sample was homogenized at 10,000 r/min for 2 min in 9 mL of 1% sulfosalicylic acid (m/V) and centrifuged at 10,000 r/min for 15 min at 4 °C. The supernatant was then adjusted to pH 2.2 with 2 mol/L HCl. Finally, the sample was analyzed with the amino acid analyzer [46]. The analysis was conducted with the following conditions: chromatographic column = XBridge-C18-T (5 μm, 4.6 mm × 250 mm); column temperature = 25 °C; mobile phase A = phosphate saline buffer (1.40 mmol/L C_16_H_37_NO_4_S; 0.01 mol/L K_2_HPO_4_; pH 3.2); mobile phase B = methanol; flow rate = 1 mL/min; injection volume = 10 μL; isocratic elution; A:B = 98:2, 25 min.

### 2.7. Fatty Acid Composition

Detection protocols drew from modified procedures documented by Figueiredo et al. [47], adapted to the characteristics of the samples used in this study. Exactly 1.0000 g of ground muscle sample was accurately weighed and placed into a clean centrifuge tube. A 4.0 mL aliquot of 1.5 M sodium hydroxide–methanol solution was added, followed by an appropriate amount of homogenate. The mixture was thoroughly vortexed to ensure complete contact between the sample and reagents. The tube was then placed in an ultrasonic bath and sonicated at room temperature for 15 min to facilitate the full release and saponification of fatty acids from the sample. After completion of the alkaline saponification, 4.0 mL of 1.5 M sulfuric acid-methanol solution was slowly added to the tube, which was gently inverted to mix uniformly before being returned to the ultrasonic bath for an additional 12 min to achieve fatty acid methylation. Upon completion of methylation, 2 mL of n-hexane (Yonghua Chemical Co., Ltd., Suzhou, Jiangsu, China) was added, and the mixture was vortexed for 30 s to fully dissolve fatty acid methyl esters (FAMEs) in the n-hexane phase. The tube was then centrifuged at 3000 rpm for 5 min to achieve phase separation. After centrifugation, the upper n-hexane phase (containing FAMEs) was carefully pipetted into a clean sample vial, sealed, and submitted for analysis using an automated gas chromatograph (GC). Chromatographic separation of FAMEs was conducted on an Agilent Technologies (Santa Clara, CA, USA) GC system equipped with a 5977A mass selective detector (MSD), using an HP-88 capillary column (60 m × 0.25 mm × 0.25 μm, Agilent Technologies, Inc., Santa Clara, California, United States). The specific GC operating conditions were set as described by Zhang et al. [48]. The column oven temperature program was as follows: the initial temperature was maintained at 130 °C for 5 min, then increased at 4 °C/min to 240 °C, which was maintained for 30 min. The carrier gas used was nitrogen with a flow rate of 0.5 mL/min. The injection volume was 1 µL at a split ratio of 10:1, and the injector and FID detector temperatures were kept at 250 °C. FAMEs were identified by matching retention times to a commercial 35-component FAME standard mixture (Supelco 37 Component FAME Mix, CRM47885, Supelco/Sigma-Aldrich, St. Louis, MO, USA; Lot No. LRAC3241). A retention-time match within ±0.05 min was required. When MS spectra were available, identification was further confirmed by mass spectral library matching (NIST, match score ≥ 80). Because the present study aimed to compare relative fatty acid profiles among treatments, fatty acids were quantified as the percentage of total identified FAMEs. No internal standard was used, and no response-factor correction or absolute quantification was performed. Results are expressed as the percentage of total identified FAMEs; unidentified peaks were excluded from the calculation.

### 2.8. Muscle Histomorphology Observation

For histomorphological analysis, the right pectoralis major (breast muscle) and the right gastrocnemius (leg muscle) were sampled from the same bird. The mid-belly of each muscle was selected, and muscle strips were cut parallel to the muscle fiber direction. Fresh muscle tissues were trimmed into uniform 0.5 cm × 0.5 cm × 0.2 cm blocks and fixed in 4% paraformaldehyde for 24 h. Gradient ethanol dehydration ranging from 70% to 100% and dual xylene clearing steps preceded paraffin embedding. Embedded tissues were sliced into 5 μm serial sections, mounted on poly L lysine slides and baked at 60 °C for half an hour to strengthen tissue adhesion. H&E staining followed standard histological workflows, including xylene dewaxing, gradient rehydration, hematoxylin nuclear staining, hydrochloric acid ethanol differentiation, tap water rinsing, eosin cytoplasmic counterstaining, secondary dehydration and xylene transparentization. Neutral resin sealed slices for long term preservation. Clear tissue fields were photographed under a LEICA DMI8 structured light microscope. For each chicken, two paraffin sections were prepared. From each section, five non-overlapping fields were randomly selected using a grid-based method, avoiding edges, folds, and damaged areas; no subjective selection of myofiber morphology was made. Thus, a total of 10 fields were analyzed per chicken. For myofiber counting, a consistent and unbiased inclusion/exclusion rule was applied: myofibers completely within the field or touching the upper and left borders were counted, whereas those touching the lower and right borders were excluded. This rule was uniformly adopted for all fields to minimize selection bias. Myofibers with clear cross-sectional profiles were measured. At least 200 myofibers were measured per chicken, corresponding to an average of 20–30 myofibers per field across the 10 fields. Both myofiber density (number per field) and cross-sectional area were derived from the same set of captured images and the same myofiber population. Cross-sectional area and diameter were analyzed with Image (version 2.18.0). The examiner was blinded to sample grouping via anonymized IDs. For each chicken, all measurements were averaged to obtain a single individual mean, which served as the biological replicate for subsequent statistical analyses; individual myofiber or single-field values were not used for between-group comparisons.

### 2.9. Transcriptomic Analysis and Quantitative Real-Time PCR Verification

For RNA-seq, two birds per pen were pooled to form one library per pen, yielding 6 libraries per treatment and 24 libraries in total. The pen was the experimental unit (*n* = 6 pen-level replicates per treatment). Library preparation and sequencing were performed in 5 batches. Samples from different treatment groups were randomized across batches to avoid confounding batch effects with treatment. Total RNA was isolated from 100 mg Danzhou chicken breast muscle samples using Trizol reagent (Invitrogen, Carlsbad, CA, USA) per the manufacturer’s instructions. RNA purity (A260/A280 ratio 1.8~2.0) and integrity were verified by Nanodrop nucleic acid detector and agarose gel electrophoresis, respectively. Sequencing libraries were constructed with the NEBNext^®^ Ultra™ RNA Library Prep Kit for Illumina^®^ (NEB, Ipswich, MA, USA). mRNA was enriched with Oligo (dT) magnetic beads, fragmented to 200~300 bp, and reverse-transcribed to double-stranded cDNA. Following end repair, adapter ligation, and PCR amplification, library quality was assessed by Agilent 2100 Bioanalyzer (Agilent Technologies, Santa Clara, CA, USA), with fragment size 200~300 bp and concentration ≥10 ng/μL. Transcriptome sequencing was performed on the Illumina NovaSeq platform (Illumina, Inc., San Diego, CA, USA) to generate 150 bp paired-end (PE) raw reads. FASTQ-formatted raw reads underwent quality control to remove reads containing adapters, reads with a high N content (N > 5%), and low-quality reads (Phred quality score Q < 20 for >50% of the read). Filtered reads were further processed using Cutadapt (v1.9.1) to obtain clean reads, and read quality was assessed using FastQC (v0.11.9). Clean reads were aligned to the chicken reference genome (GRCg6a) using HISAT2 (v2.2.1), and SAM files were converted to BAM format using SAMtools(v1.17). Gene expression was quantified as fragments per kilobase of transcript per million mapped reads (FPKM) using featureCounts (v1.5.0-p3). Raw read counts were generated using featureCounts (v2.0.1) and used as input for DESeq2. FPKM values were calculated only for descriptive visualization and were not used for differential expression analysis. Differentially expressed genes (DEGs) between groups were screened with the DESeq2 R package (v1.16.1), using |log_2_(fold change)| ≥ 1 and adjusted *p*-value (padj) < 0.05 as criteria. Read depth and mapping statistics for each RNA-seq sample are provided in Supplementary Table S2. GO (Gene Ontology) and KEGG (Kyoto Encyclopedia of Genes and Genomes) enrichment analyses of DEGs were conducted with the clusterProfiler R package(v4.10.0), with padj < 0.05. All bioinformatics analyses were performed on the NovoMagic cloud platform (https://magic.novogene.com/).

For RT-qPCR verification, Trizol Up reagent extracted RNA from breast and leg muscle samples ranging from 1.5 to 2 g. RNA integrity and purity were rechecked through agarose electrophoresis and NanoPhotometer detection, only samples with A260/A280 values between 1.8 and 2.1 proceeded to reverse transcription. Amplification was performed on a StepOne Plus PCR instrument(Applied Biosystems, Foster City, CA, USA) with SYBR Green master mix; each 20 μL reaction contained cDNA, forward and reverse primers, ROX reference dye and nuclease-free water. Thermal cycling included three minutes initial denaturation, 40 cycles of denaturation annealing extension and terminal melting curve scanning to confirm amplicon specificity. Standard curves confirmed primer amplification efficiency between 95% and 105%. Six pen-level biological replicates per treatment (two birds per pen were pooled/averaged) with three technical replicates each were analyzed. *β actin* and *GAPDH* served as dual reference genes, and their expression stability was validated by geNorm. The 2^−ΔΔCt^ algorithm calculated relative mRNA abundance, with the control group set as the reference calibrator. All measured indicators are listed in Table 1.

**Table 1:** Primers used for quantitative RT-qPCR.

Gene Name	Primer Sequence (5′-3′)	Length of Product (bp)	Accession Number
*ABCA1*	F: CTCGGGAGAAGTGGAGCGAA	251	NM_204145.3
	R: GGCCAAGCAACTTCGATCAG		
*IRS2*	F: GCCTCAACTACATCGCCGT	216	XM_015277882.4
	R: TCCAAGCATAGGTTTTCTCCTGG		
*IGF1*	F: TACCTTGGCCTGTGTTTGCT	170	NM_001004384.3
	R: CCCTTGTGGTGTAAGCGTCT		
*MyoG*	F: AAGGGGAGAAGTGTGATGGC	177	NM_204184.2
	R: ACAGCGGTCAGTAGGAAAGC		
*AKT1*	F: GAGAAGGGGAGGAGGAGCC	223	NM_001396387.1
	R: GGAGCGGGTCACTTACATGG		
*FOXO1*	F: TGCTATGAACCCGTATGCCC	246	NM_204328.2
	R: ACCCAACTGTGTGTGGTTGT		
*ACTB*	F: GTGGATCAGCAAGCAGGAGT	182	NM_205518
	R: ATCCTGAGTCAAGCGCCAAA		
*GAPDH*	F: GGGCACGCCATCACTATCTT	187	NM_204305
	R: TCACAAACATGGGGGCATCA		

*ABCA1*, *ATP binding cassette subfamily A member 1*; *IRS2*, *insulin receptor substrate 2*; *IGF1*, *insulin-like growth factor 1*; *MyoG*, *myogenin; AKT1*, *AKT serine/threonine kinase 1*; *FOXO1*, *forkhead box O1*; *ACTB*, *Actin Beta*; *GAPDH*, *Glyceraldehyde-3-Phosphate Dehydrogenase*.

### 2.10. Metabolomics Analysis

Approximately 50 mg of Danzhou chicken breast muscle (impurities removed, cut into 1–2 mm pieces) was accurately weighed into 2 mL sterile centrifuge tubes. A 600 μL aliquot of chromatographic grade methanol containing 4 ppm internal standard 2-chloro-L-phenylalanine was added, and the mixture was vortexed at 2500 rpm for 30 s. After adding one 5 mm sterile stainless-steel bead, samples were homogenized at 50 Hz for 120 s, followed by ultrasonic extraction at room temperature (200 W, 3 s on/3 s off) for 10 min. Samples were centrifuged at 12,000 rpm and 4 °C for 10 min. The supernatant was vacuum-filtered through a 0.22 μm organic phase filter (13 mm diameter), and the filtrate was transferred to LC-MS vials (with inner liners), sealed, and stored at 4 °C for LC-MS/MS analysis. Three QC samples (equal volumes of all samples) were prepared to assess reproducibility and instrument stability. Pooled QC samples were prepared by mixing equal volumes of all samples. QC samples were injected at the beginning of the run, after every 10 samples, and at the end of the run. The injection sequence was randomized, and solvent blanks were injected between batches to monitor carryover. Chromatographic separation was performed on a Hypersil GOLD C18 column (2.1 × 100 mm, 1.9 μm; Thermo Scientific, Shanghai, China) at 40 °C (±0.1 °C). Mobile phases were 0.1% (*v*/*v*) formic acid in ultrapure water (A, MS grade) and 0.1% (*v*/*v*) formic acid in acetonitrile (B, chromatographic grade), with a constant flow rate of 0.3 mL/min and injection volume of 5 μL. Gradient elution: 0–1.0 min (5% B), 1.0–12.0 min (5–95% B linear), 12.0–14.0 min (95% B), 14.0–14.1 min (95–5% B), 14.1–16.0 min (5% B re-equilibration). MS detection was conducted with ESI (positive/negative modes, *m*/*z* 50–1000): ion source voltage (3.5 kV positive, 3.0 kV negative), ion source temperature 350 °C, sheath gas 35 arb, auxiliary gas 10 arb, capillary temperature 300 °C. Data-dependent MS/MS acquisition was performed with a stepped normalized collision energy of 20, 35, 50% (NCE), an isolation window of 1.0 Da, and a dynamic exclusion time of 10 s. MS and MS/MS data were acquired on a Thermo Q Exactive HF-X mass spectrometer operated at a resolution of 120,000 (full MS) and 15,000 (MS/MS) in positive and negative ion modes. Raw data were converted to mzXML via ProteoWizard MSConvert (v3.0.18328). Data preprocessing was conducted using the XCMS software package (v4.6.3), with the centWave algorithm (ppm = 20, peakwidth = c (5, 30)) employed for peak detection, the obiwarp algorithm for peak alignment, and the fillPeaks function for supplementing missing peaks. SVR-based correction with QC sample RSD > 30% metabolite exclusion were applied. SIMCA 14.1 was used for statistical analysis. Preprocessed data (centralized, Pareto-scaled) were used to construct OPLS-DA models (acceptable if R^2^X > 0.5, R^2^Y > 0.8, Q^2^ > 0.5). OPLS-DA was performed for exploratory visualization of group separation. Model parameters were reported as R^2^X(cum), R^2^Y(cum), and Q^2^(cum). Given the limited predictive ability of the OPLS-DA model (Q^2^ < 0.5), differential metabolites were primarily identified by univariate analysis: Shapiro–Wilk normality test, Levene’s test for homogeneity of variance, followed by Student’s *t*-test or Wilcoxon rank-sum test as appropriate. *p*-values were adjusted using the Benjamini–Hochberg procedure, and metabolites with FDR < 0.05 and |log_2_FC| ≥ 0.5 were considered significantly different. VIP > 1 from OPLS-DA was used only as an auxiliary criterion for ranking, not as a primary screening threshold. Model quality was assessed by R^2^X(cum), R^2^Y(cum), and Q^2^(cum), with Q^2^ > 0.5 considered acceptable for predictive interpretation. When Q^2^ was below this threshold, the model was treated as exploratory only, and VIP was not used as a primary selection criterion. Metabolites were annotated via HMDB and Metlin.

### 2.11. Statistical Analysis

All numerical data were analyzed using SPSS 26.0 (IBM, Armonk, NY, USA) and visualized using GraphPad Prism 8.0. The Shapiro–Wilk test for normality and Levene’s test for homogeneity of variance were performed before parametric analysis. Data satisfying both assumptions were analyzed using one-way ANOVA, followed by Duncan’s multiple-range test when the overall treatment effect was significant. Results are presented as the mean ± standard error of the mean, and statistical significance was defined as *p* < 0.05. Orthogonal polynomial contrasts were used to assess linear and quadratic responses to graded NCG supplementation. For integrated correlation analysis, Spearman correlation was used; *p*-values were adjusted by the Benjamini–Hochberg procedure; |r| > 0.8 and FDR < 0.05 were considered significant; coefficients and adjusted *p*-values are reported in Supplementary Tables S3–S5. No formal dose-optimization model was fitted. Therefore, conclusions regarding dose effects are restricted to the doses tested, and no true optimum or biological threshold can be established. The model was Yij = μ + Ti + eij, where Yij is the observed value, μ is the overall mean, Ti is the fixed effect of treatment, and eij is the random error. For tissue outcomes, the pen was the experimental unit. Two birds per pen were sampled, and values were averaged within each pen to obtain one independent observation per pen. Six pens per treatment were used (*n* = 6 independent replicates per treatment). For RNA-seq, two birds per pen were pooled into one library, and the pen was the experimental unit (*n* = 6 pen-level replicates per treatment). For the four treatment groups, the error degrees of freedom were E = N − k = k(n − 1) = 4 × (6 − 1) = 20, which falls within the acceptable range of 10–20 recommended by Arifin and Zahiruddin [49].

## 3. Results

### 3.1. Growth Performance

Mortality was recorded for each treatment: CON, 0/120; N1, 0/120; N2, 0/120; N3, 2/120 (1.7%). FBW, ADG, and FCR were significantly affected (*p* = 0.0221, 0.0221, and 0.0074, respectively) by graded levels of dietary NCG. FBW and ADG exhibited a quadratic resp onse (*p* = 0.0103 and *p* = 0.0103, respectively), whereas FCR showed both linear (*p* = 0.0074) and quadratic (*p* = 0.0049) responses. No significant differences (*p* = 0.4197) in ADFI were detected among the treatment groups (Table 2).

### 3.2. Muscle Composition

Crude protein content in both breast muscle and leg muscle was significantly affected (*p* = 0.0021, 0.0242, respectively) by graded levels of dietary NCG, showing a linear response (*p* = 0.0005) in breast muscle and a quadratic response (*p* = 0.0077) in leg muscle. No significant differences (*p* > 0.05) were observed in moisture, fat, ash or IMP (*p* = 0.9817, *p* = 0.9255, *p* = 0.9936, *p* = 0.4443) (Table 3).

### 3.3. Free Amino Acid Profile

Graded dietary levels of NCG significantly affected the contents of phenylalanine, arginine, cysteine, and methionine (*p* = 0.0259, 0.0127, 0.0395, and 0.0488, respectively). The contents of phenylalanine, cysteine, and methionine displayed quadratic responses (*p* = 0.0037, 0.0077, 0.0240, respectively), while arginine content exhibited both linear (*p* = 0.0098) and quadratic (*p* = 0.0286) responses. Notably, total amino acids also exhibited a quadratic response (*p* = 0.0260) (Table 4).

Graded dietary levels of NCG significantly affected the contents of arginine and methionine (*p* = 0.0234 and 0.0305, respectively). Methionine content displayed a quadratic response (*p* = 0.0091), whereas arginine content exhibited both linear (*p* = 0.0155) and quadratic (*p* = 0.0461) responses (Table 5).

### 3.4. Fatty Acid Composition

The contents of arachidonic acid (C20:4n6), docosahexaenoic acid (C22:6n3), and total monounsaturated fatty acids (∑MUFA) in breast muscle were significantly affected (*p* = 0.0117, 0.0045, 0.0430, respectively) by graded dietary levels of NCG, each of which exhibited a quadratic response (*p* = 0.0038, 0.0033, 0.0097, respectively) (Table 6). All fatty acid data are expressed as percentages of total identified FAMEs.

Graded dietary levels of NCG significantly influenced the contents of arachidonic acid (C20:4n6) and docosahexaenoic acid (C22:6n3) in leg muscle (*p* = 0.0076 and 0.0074, respectively). Specifically, the former exhibited both linear and quadratic responses (*p* = 0.0120, 0.0096, respectively), whereas the latter displayed a linear response (*p* = 0.0034) (Table 7).

### 3.5. Muscle Fiber Traits

**Figure 1:**
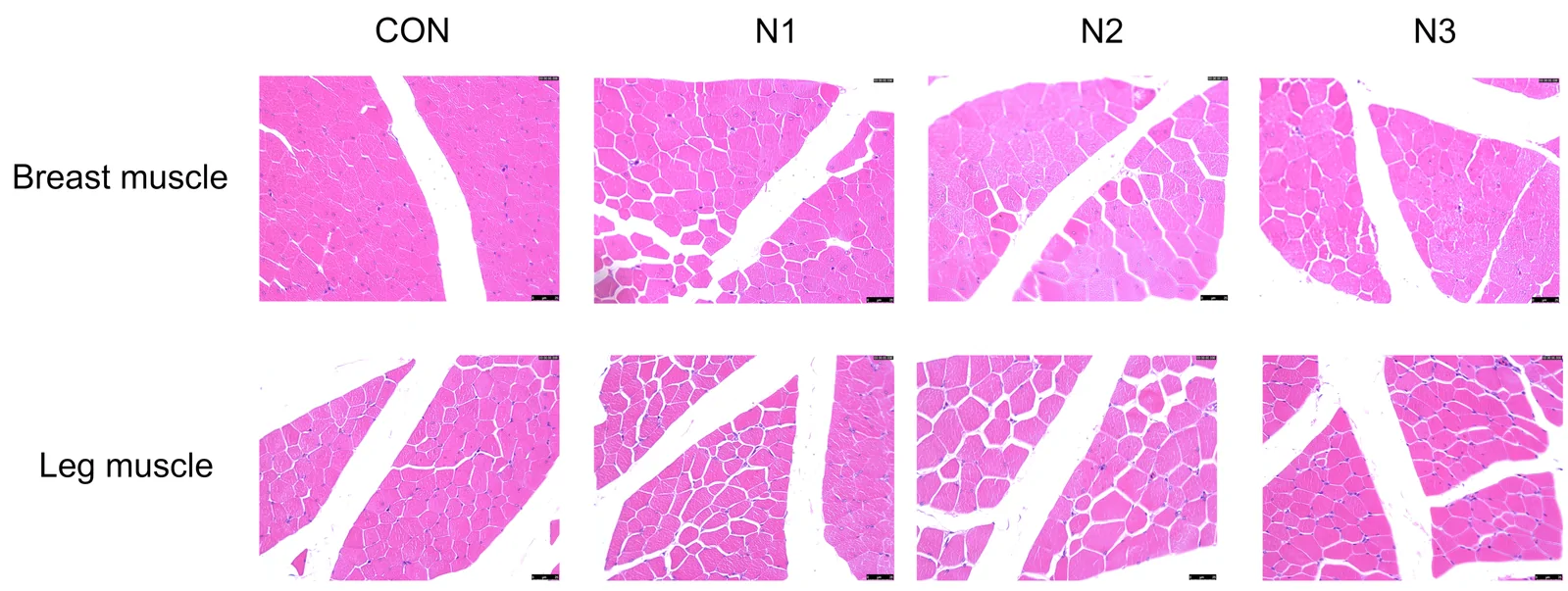
Effects of dietary NCG on the muscle fiber traits of Danzhou chickens. CON (*n* = 6), basal diet; N1 (*n* = 6), basal diet plus 400 mg/kg NCG; N2 (*n* = 6), basal diet plus 800 mg/kg NCG; N3 (*n* = 6), basal diet plus 1200 mg/kg NCG.

Graded dietary levels of NCG significantly affected (*p* = 0.0360) muscle fiber density in breast muscle, which exhibited a quadratic response (*p* = 0.0210). In leg muscle, muscle fiber area, muscle Feret diameter, and muscle fiber density were influenced (*p* =0.0464, 0.0168, 0.0366, respectively) by graded dietary NCG; the former two parameters showed quadratic responses (*p* = 0.0129 and 0.0033, respectively), whereas the latter exhibited both linear and quadratic responses (*p* = 0.0305, and 0.0402, respectively) (Table 8, Figure 1).

### 3.6. Muscle Transcriptomic Analysis

**Figure 2:**
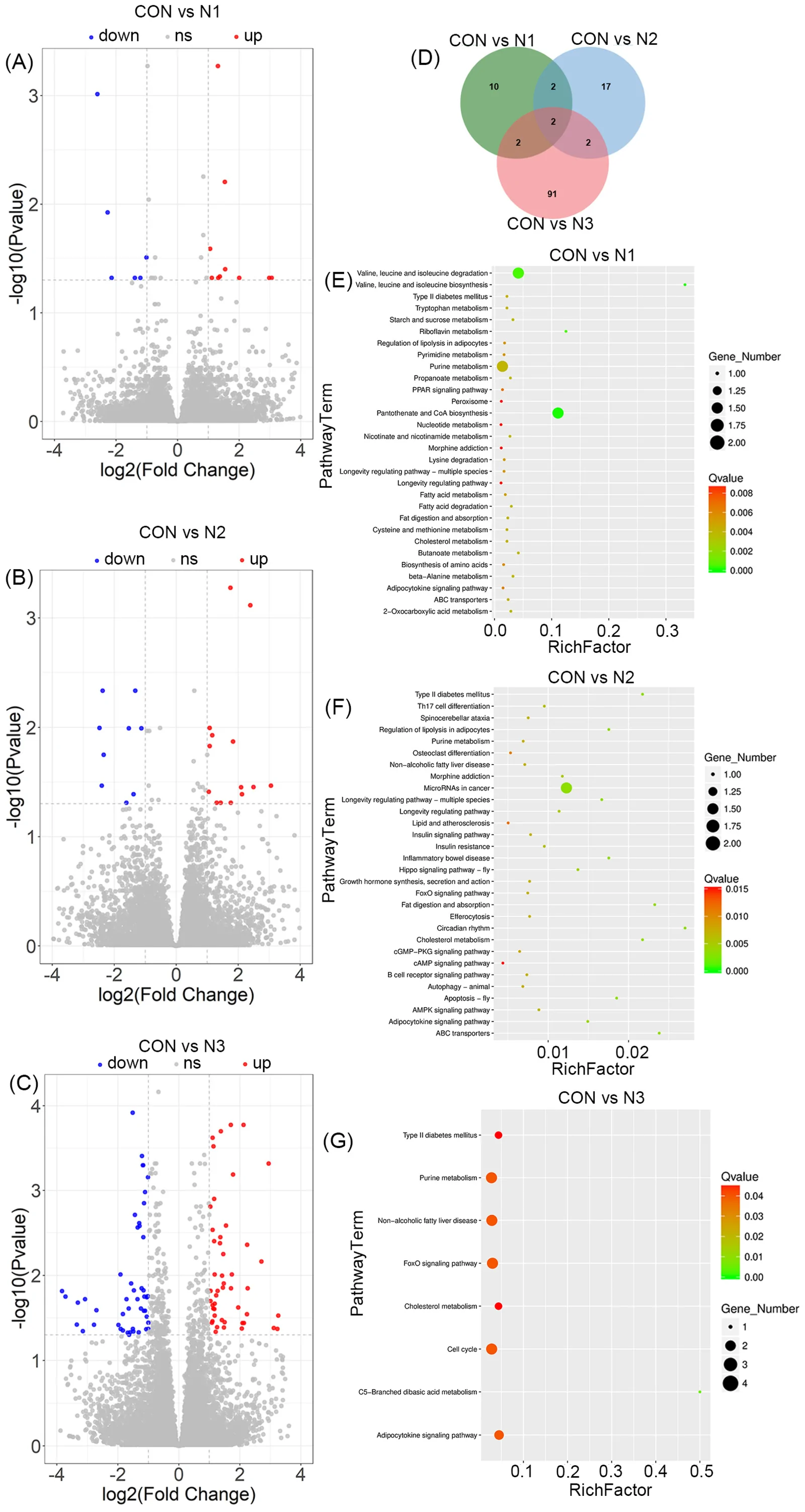
Analysis of differentially expressed genes (DEGs) in the transcriptome. (**A**–**C**), volcano plots of DEGs in each group; (**D**), Venn diagram of DEGs among different groups; (**E**–**G**), KEGG enrichment pathway diagrams of DEGs in each group. CON (*n* = 6), basal diet; N1 (*n* = 6), basal diet plus 400 mg/kg NCG; N2 (*n* = 6), basal diet plus 800 mg/kg NCG; N3 (*n* = 6), basal diet plus 1200 mg/kg NCG.

DEGs were defined as genes with Benjamini–Hochberg adjusted *p* < 0.05 and |log2FC| ≥ 1. A total of 24,397 genes in the transcriptome database were functionally annotated. Volcano plot analysis demonstrated that, relative to the control group, the N1 group exhibited 6 downregulated and 10 upregulated genes; the N2 group showed 9 downregulated and 14 upregulated genes; and the N3 group displayed 49 downregulated and 49 upregulated genes (Figure 2A–C). An unsupervised PCA plot based on variance-stabilizing transformed normalized counts is provided in Figure S1. Samples from the same treatment group clustered together, and no obvious batch-driven separation was observed. Venn diagram analysis of overlapping differentially expressed genes (DEGs) identified 3 common DEGs between CON vs. N1 and CON vs. N2, as well as 3 common DEGs between CON vs. N1 and CON vs. N3, while 13 common DEGs were detected between CON vs. N2 and CON vs. N3. Among these, *IRS2* and *ABCA1* were shared annotated DEGs shared across all three pairwise comparisons (Figure 2D).

Subsequently, GO and KEGG enrichment analyses were conducted to systematically explore the functions of DEGs in the muscle tissue of Danzhou chickens and their regulatory roles in lipid metabolism. GO enrichment analysis revealed that DEGs were predominantly enriched in terms related to carbohydrate metabolism, energy metabolism, and amino acid metabolism. In total, 16, 23, and 98 DEGs were identified in the N1, N2, and N3 groups compared with the control group, respectively. KEGG enrichment analysis further annotated 52, 32, and 140 signaling pathways in the corresponding groups, and dietary NCG supplementation significantly enriched pathways including cholesterol metabolism, the adipocytokine signaling pathway, the FoxO signaling pathway, and purine metabolism (Figure 2E–G; Supplementary Figure S2A–C). Adjusted *p*-values and gene counts for each enriched term are reported in Supplementary Table S6. Because the DEG sets for N1 and N2 were small, enrichment results should be interpreted cautiously.

### 3.7. Metabolomics Analysis

**Figure 3:**
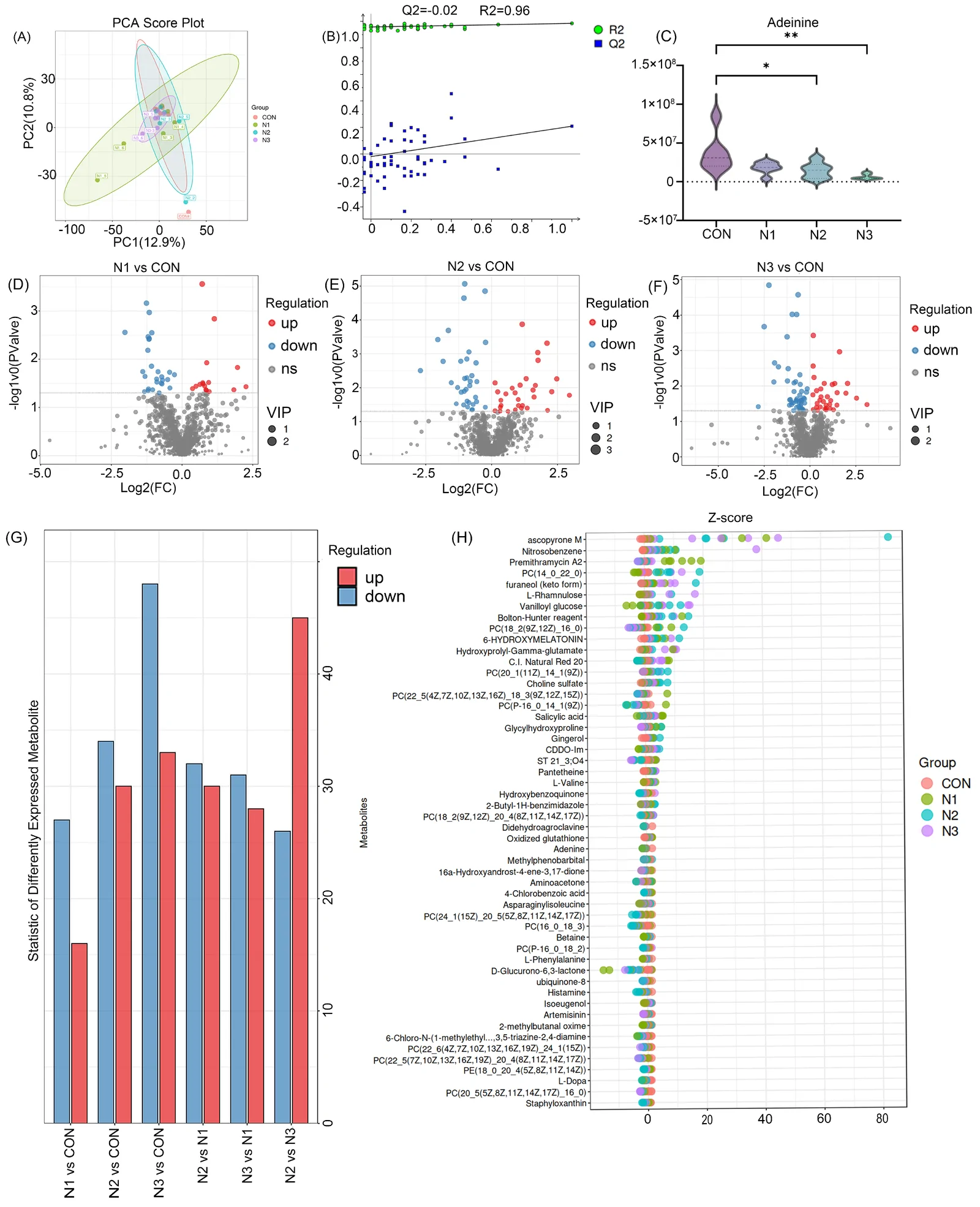
Results of differential metabolite screening. (**A**), Metabolomics PCA plot; (**B**), permutation test plot of the OPLS-DA model; (**C**), relative abundance of adenine; (**D**–**F**), volcano plots of differential metabolites in each group; (**G**), differential metabolite count plot; (**H**), Z-score plot. CON (*n* = 6), basal diet; N1 (*n* = 6), basal diet plus 400 mg/kg NCG; N2 (*n* = 6), basal diet plus 800 mg/kg NCG; N3 (*n* = 6), basal diet plus 1200 mg/kg NCG. * represents *p* < 0.05; ** represents *p* < 0.01.

Metabolomics analysis revealed differences in metabolites among the CON, N1, N2, and N3 groups. In the PCA score plot, samples from the four groups were represented by colored symbols, with moderate dispersion along the first principal component (PC1) and second principal component (PC2), Samples from the same treatment group clustered together, and no obvious batch-driven separation was observed. (Figure 3A). The original OPLS-DA model had R^2^X(cum) = 0.184, R^2^Y(cum) = 0.985, and Q^2^(cum) = 0.211, with one predictive and one orthogonal component. The high R^2^Y but low Q^2^ indicated limited cross-validated predictive ability, suggesting that the model should be considered exploratory. Permutation testing (200 permutations) gave an R^2^ intercept of 0.96 and a Q^2^ intercept of -0.03; the negative Q^2^ intercept indicated no severe overfitting (Figure 3B). Given the limited Q^2^ of the original model, differential metabolites were re-selected using univariate FDR-controlled statistics, and VIP was used only as a supplementary ranking measure. All differential metabolites are reported with FDR, -log10(*p*.value), VIP, and identification confidence level in Supplementary Table S7.

**Figure 4:**
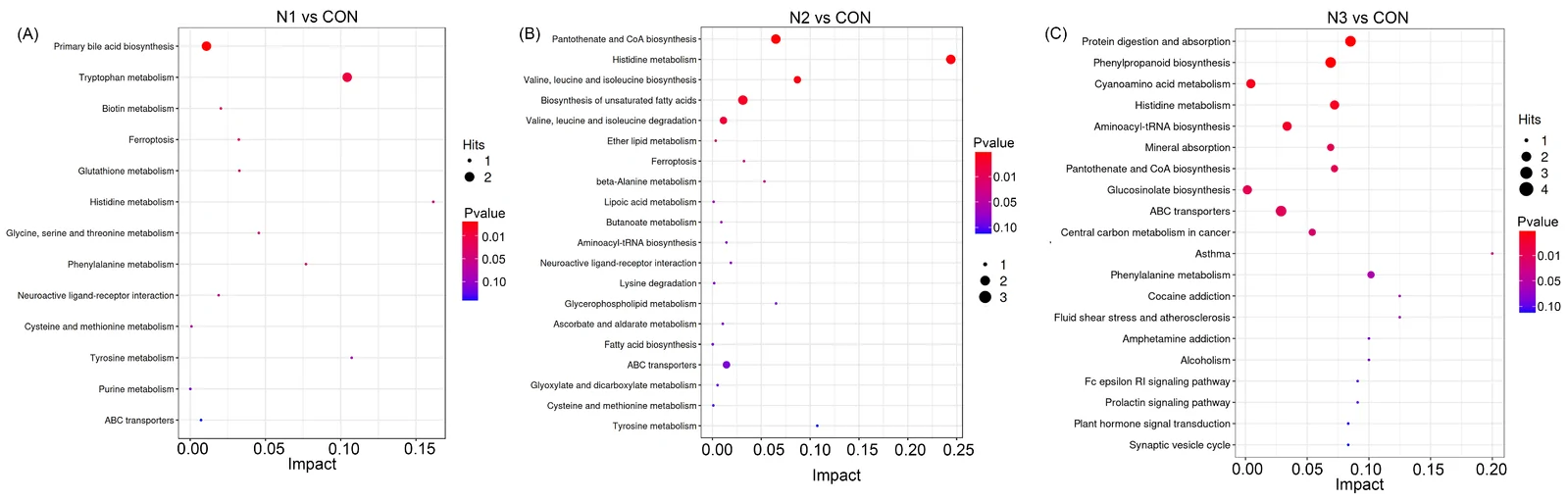
Bubble plots of metabolite pathway enrichment. (**A**–**C**), KEGG enrichment pathway bubble plots of each group. CON (*n* = 6), basal diet; N1 (*n* = 6), basal diet plus 400 mg/kg NCG; N2 (*n* = 6), basal diet plus 800 mg/kg NCG; N3 (*n* = 6), basal diet plus 1200 mg/kg NCG.

Differential metabolites were screened via a volcano plot and visualized using a Z-score plot (Figure 3D–F,H). Inter-group difference analysis identified 43 differential metabolites between the N1 and CON groups (16 up-regulated and 27 down-regulated), 64 differential metabolites between the N2 and CON groups (30 up-regulated and 34 down-regulated), and 81 differential metabolites between the N3 and CON groups (33 up-regulated and 48 down-regulated) (Figure 3G). Among them, adenine showed lower relative abundance in the NCG groups than in the CON group (Figure 3C). These differential metabolites were closely associated with nutrient metabolism. Further KEGG enrichment analysis identified four key pathways: Purine metabolism, ABC transporters, Cysteine and methionine metabolism, and Tryptophan metabolism (Figure 4A–C).

### 3.8. Quantitative Real-Time PCR Analysis

**Figure 5:**
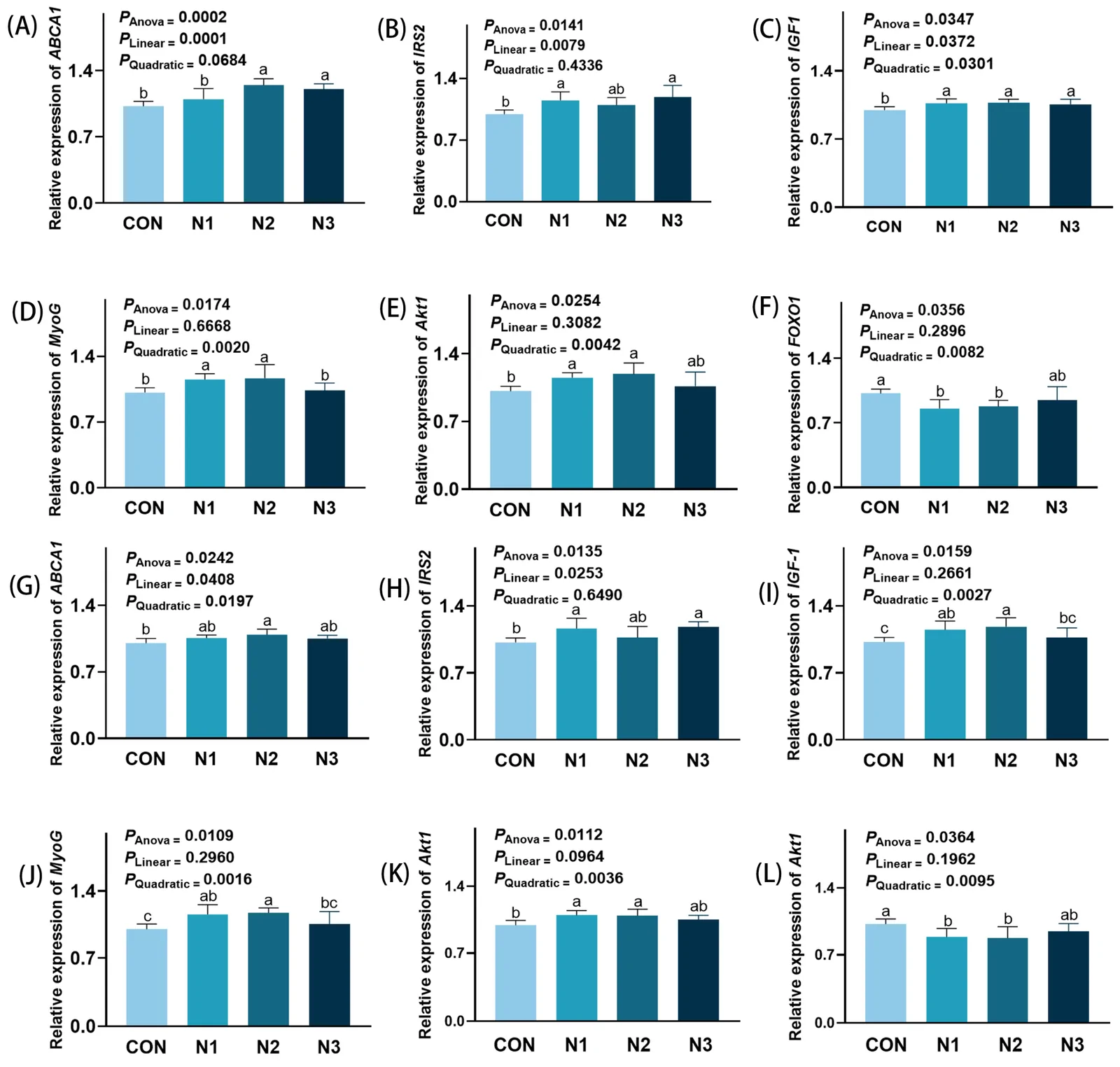
Effect of NCG on the related gene expression of breast and leg muscles in Danzhou Chickens. (**A**–**F**) represent the expression levels of related genes in breast muscle samples, while (**G**–**L**) represent those in leg muscle samples. *ABCA1*, *ATP binding cassette subfamily A member 1*; *IRS2*, *insulin receptor substrate 2*; *IGF1*, *Insulin-Like Growth Factor 1*; *MyoG, myogenin; AKT1, AKT serine/threonine kinase 1*; *FOXO1*, *forkhead box O1*. CON (*n* = 6), basal diet; N1 (*n* = 6), basal diet plus 400 mg/kg NCG; N2 (*n* = 6), basal diet plus 800 mg/kg NCG; N3 (*n* = 6), basal diet plus 1200 mg/kg NCG. ^a, b, c^ Means in the row without common superscripts are significant difference (*p* < 0.05).

Based on the preceding analysis, two representative genes (*ABCA1* and *IRS2*), together with growth-related genes (*IGF1* and *MyoG*) and key pathway genes (*AKT1* and *FOXO1*), were selected for quantitative real-time polymerase chain reaction (qRT-PCR) to verify their expression at the mRNA level. (Figure 5) In the breast muscles, graded levels of dietary NCG significantly influenced the relative expression of *ABCA1*, *IRS2*, *IGF1*, *MyoG*, *AKT1*, and *FOXO1* (*p* = 0.0002, 0.0141, 0.0347, 0.0174, 0.0254, and 0.0356, respectively). The relative expression of *ABCA1* and *IRS2* responded linearly to dietary NCG (*p* = 0.0001 and 0.0079, respectively), while *MyoG*, *AKT1*, and *FOXO1* exhibited quadratic responses (*p* = 0.0020, 0.0042, and 0.0082, respectively). In contrast, *IGF1* expression showed both linear (*p* = 0.0372) and quadratic (*p* = 0.0301) responses to the graded NCG levels. (Figure 5A–F) In the leg muscles, dietary NCG at graded concentrations also significantly affected the relative expression of these six genes (*ABCA1*, *p* = 0.0242; *IRS2*, *p* = 0.0135; *IGF1*, *p* = 0.0159; *MyoG*, *p* = 0.0109; *AKT1*, *p* = 0.0112; *FOXO1*, *p* = 0.0364). *IRS2* expression responded linearly to dietary NCG (*p* = 0.0253), whereas *IGF1*, *MyoG*, *AKT1*, and *FOXO1* displayed quadratic responses (*p* = 0.0027, 0.0016, 0.036, and 0.0095, respectively). *ABCA1*, on the other hand, showed both linear (*p* = 0.0408) and quadratic (*p* = 0.0197) responses to the graded NCG treatments (Figure 5G–L).

### 3.9. Integrated Analysis of Transcriptomic and Metabolomic Data

**Figure 6:**
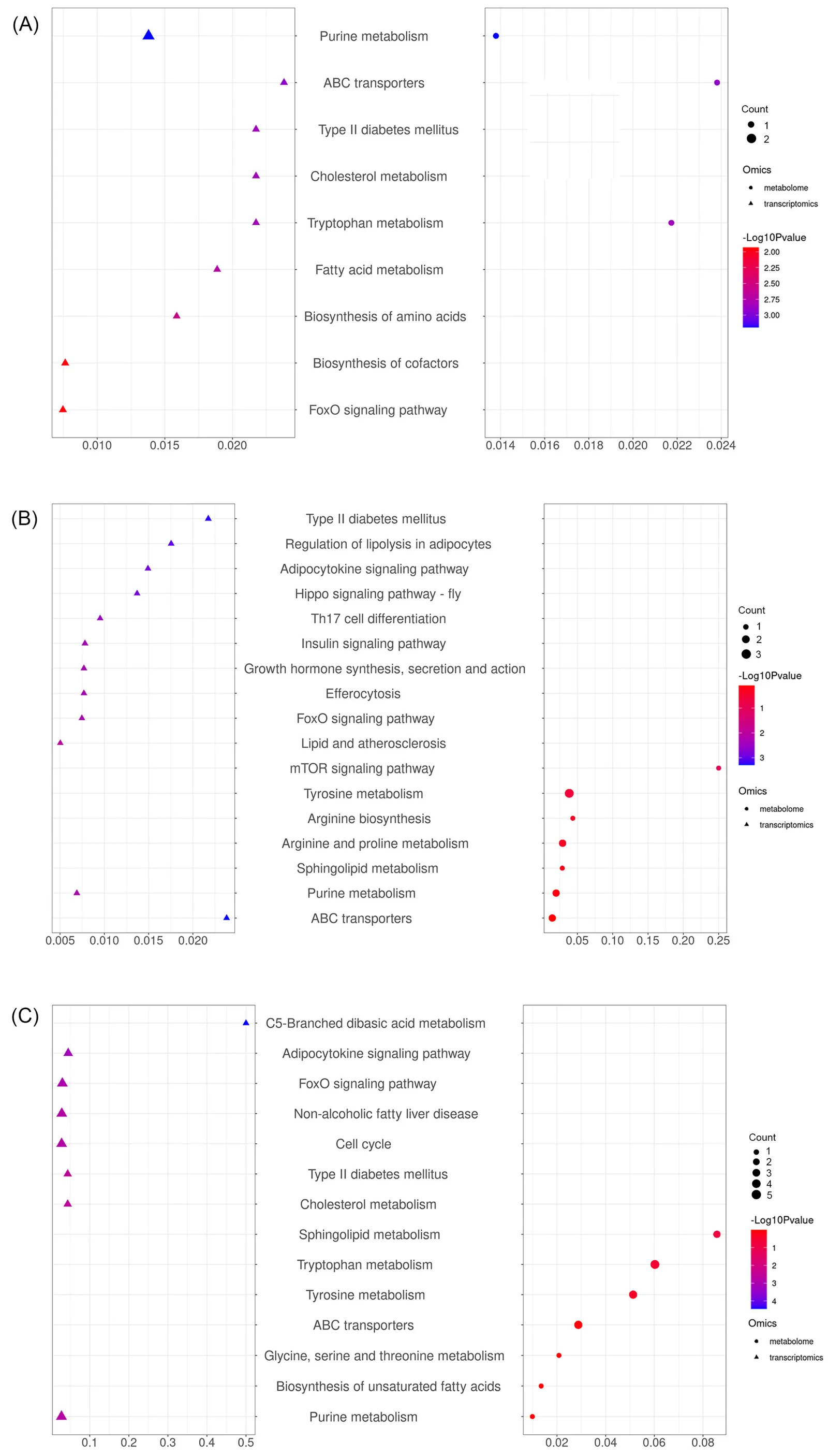
Integrated bubble plots of transcriptomic and metabolomic analyses. (**A**), Integrated bubble plot of transcriptomic DEGs and metabolomic profiles between CON and N1; (**B**), integrated bubble plot of transcriptomic DEGs and metabolomic profiles between CON and N2; (**C**), integrated bubble plot of transcriptomic and metabolomic profiles between CON and N3. CON (*n* = 6), basal diet; N1 (*n* = 6), basal diet plus 400 mg/kg NCG; N2 (*n* = 6), basal diet plus 800 mg/kg NCG; N3 (*n* = 6), basal diet plus 1200 mg/kg NCG.

Correlation analysis revealed significant associations between differential metabolites and differentially expressed genes (Figure S3A–C, Tables S3–S5). In the CON vs. N1 comparison, differential metabolites were predominantly involved in amino acid metabolism and antioxidant processes; for instance, L-Dopa was significantly positively correlated with genes including *ABCA1* and *ENPP1*. In the CON vs. N2 comparison, differential metabolites were dominated by phospholipids (e.g., various phosphatidylcholines, PCs), which showed strong correlations with corresponding genes, suggesting altered lipid metabolism and cell membrane remodeling. In the CON vs. N3 comparison, differential metabolites participated in purine metabolism, among which adenine was significantly correlated with genes including *IRS2*. Collectively, distinct clustering patterns were observed across different modules, indicating exploratory associations between differentially expressed genes and metabolites. Furthermore, comparative integrated analysis identified overlapping enriched pathways from transcriptomic and metabolomic datasets. Notably, purine metabolism was significantly enriched in both transcriptome- and metabolome-derived KEGG enrichment analyses (Figure 6A–C).

## 4. Discussion

This study provides the first multi-omics evidence that dietary NCG modulates muscle growth, nutrient composition, and metabolic profiles in Danzhou chickens. Consistent dose-dependent effects were observed: 400–800 mg/kg NCG increased ADG and reduced FCR without altering ADFI, improved muscle amino acid and fatty acid profiles, and enlarged muscle fiber cross-sectional area, whereas 1200 mg/kg high-dose NCG failed to further boost growth performance. These data indicate NCG improves growth efficiency by elevating nutrient utilization and inducing muscle fiber hypertrophy, yet its biological function is restricted by host metabolic networks.

Our data confirmed that NCG improved weight gain without changing voluntary feed intake, a phenotypic outcome consistent with previous studies verifying NCG’s capacity to boost nutrient deposition efficiency rather than stimulating feed consumption [50]. As a rate-limiting activator of endogenous arginine biosynthesis, NCG effectively elevates circulatory and tissue arginine concentrations in vivo [22,23]. NCG-mediated arginine accumulation may act as a critical upstream signal triggering anabolic metabolism in the body. Consistent results from transcriptomic analysis and qPCR validation further revealed significantly upregulated expression of insulin receptor substrate 2 (*IRS2*). *IRS2* functions as a central molecule linking insulin/IGF-1 signaling to the downstream PI3K/Akt pathway, and its enhanced expression in breast and leg muscles can improve the sensitivity and responsiveness of muscle cells to growth factors [51,52], thereby providing a molecular basis for amplifying anabolic signals and promoting muscle growth. Meanwhile, muscle crude protein concentration and free amino acid concentrations, including arginine and methionine, were increased by NCG. However, because muscle weights, total protein mass, and protein synthesis/degradation rates were not measured, these data indicate that NCG may affect protein metabolism rather than directly demonstrating enhanced protein deposition [53]. The changing trend of IMP content in muscle suggested that NCG may indirectly enhance the synthesis of flavor-related compounds by regulating the purine metabolism pathway, thus improving the flavor of chicken meat [54].

The upregulation of *IRS2* expression was consistent with activation of the anabolic PI3K/Akt signaling pathway [51,52]. qPCR verification in the present study further confirmed corresponding changes in the gene expression levels of *IRS2*, *Akt1*, and *FoxO1*, and provided molecular evidence associated with this pathway, but did not demonstrate functional activation. At the signal transduction level, *Akt*, a core kinase governing cell growth, promotes the initiation of protein translation and thereby enhances protein synthesis via the activation of mTORC1 [55,56]. On the other hand, it suppresses the transcriptional activity of *FoxO1* through phosphorylation, downregulating the expression of muscle atrophy-related genes including *Atrogin-1* and *MuRF*1, thus reducing protein degradation [57,58]. The significant enrichment of the *FoxO1* signaling pathway, the increased cross-sectional area of muscle fibers, and the non-activation of ubiquitination-dependent protein degradation pathways observed in this study collectively supported a synergistic regulatory mechanism of “enhanced protein synthesis and inhibited degradation”, which may promote net muscle protein deposition and induce muscle fiber hypertrophy.

Integrated analyses suggested an exploratory association with purine metabolism, but causality and its contribution to muscle hypertrophy remain to be established. This pathway was significantly enriched in both omics datasets, with enrichment levels increasing in parallel with NCG dosage. Correspondingly, multiple genes related to purine metabolism, including *PDE4D*, *PDE10A*, *PDE3A*, *GDA*, *GUCY1A2*, and *ENPP1*, were upregulated, whereas adenine abundance decreased, collectively suggesting altered pathway activity and substrate utilization [59,60]. Purine metabolism can provide ATP for energy-consuming processes such as protein synthesis and transmembrane transport [61], and nucleotide building blocks for DNA and RNA synthesis in proliferating muscle cells, including activated satellite cells, thereby supporting cell proliferation and expansion of the protein-synthesis machinery [59,62]. Combined with the growth performance data, it can be inferred that the efficient operation of the purine metabolism pathway enables the organism to fully utilize adenine, providing the material basis for rapid hypertrophic muscle growth from the perspectives of both energy metabolism and nucleic acid metabolism, thus exerting a growth-promoting effect. IMP is a determinant of umami taste in poultry meat; however, the effect of NCG on IMP was not established here. Although purine metabolism was enriched, muscle IMP concentrations did not differ significantly among treatments; sensory implications require direct evaluation.

Alterations in lipid metabolism further refine the aforementioned regulatory network. In the present study, the expression level of the cholesterol transporter gene *ABCA1* was significantly upregulated, accompanied by marked enrichment of the cholesterol metabolism pathway. As a key component of cell membranes and lipid rafts, cholesterol plays an essential role in maintaining membrane structural integrity and membrane receptor-mediated signal transduction [63,64,65]. Previous studies have confirmed that IGF-1 regulates *ABCA1* expression via the PI3K/Akt/FoxO1 signaling pathway [66]. The present results revealed that Akt signaling activated by NCG similarly upregulated *ABCA1* expression in muscle tissue. Its physiological role may not be limited to promoting cholesterol efflux, but rather involves modulating the local redistribution of cholesterol and remodeling of membrane structures to supply necessary lipid components for rapidly hypertrophying muscle fibers, while maintaining stable and efficient transmission of membrane receptor signaling platforms [67]. In addition, NCG altered muscle fatty acid composition in a muscle-specific manner. In breast muscle, total MUFA increased at 400 and 800 mg/kg, whereas total PUFA did not differ that NCG may attenuate lipid peroxidation by regulating lipid metabolism, thereby improving the oxidative stability of meat while promoting growth [68,69]. Whether these changes affect lipid peroxidation, oxidative stability, off-flavor development, or shelf life was not measured in this study and remains a hypothesis requiring direct verification. qPCR verification further confirmed corresponding changes in the expression levels of *ABCA1*, *Akt1*, and *FoxO1*, providing molecular evidence for the involvement of this pathway in the regulatory mechanism.

The present study observed a distinct non-linear dose-dependent effect in response to NCG treatment. Supplementation with a high dose of NCG at 1200 mg/kg not only failed to further improve growth performance but also exhibited inhibitory trends in several indices. This phenomenon may involve multiple regulatory mechanisms. Excessive arginine can activate arginase activity and accelerate its own catabolism, thereby reducing its effective bioavailability in vivo [70,71]. Meanwhile, surplus arginine metabolized via the NOS pathway generates excessive NO, which induces oxidative stress and impairs protein synthesis [72]. Furthermore, high-dose NCG may trigger negative feedback inhibition of *IRS2*, such as mTORC1-mediated serine phosphorylation, consequently attenuating signal transduction through the IGF-1/PI3K/Akt pathway [73]. Transcriptomic results in this study revealed a reversed expression pattern of several purine metabolism-related genes in the high-dose group, indirectly supporting the possibility of disrupted metabolic homeostasis under high NCG dosage. As a native chicken breed, Previous studies suggest that indigenous breeds may respond differently from fast-growing commercial broilers to NCG [31,71,74]. However, no direct comparative trial between Danzhou chickens and commercial broilers was conducted in this study. Therefore, the possibility that Danzhou chickens are more sensitive to NCG or have a narrower optimal range should be regarded as a hypothesis requiring direct comparative validation. These findings highlight the importance of precision nutrition in livestock and poultry production, suggesting that nutritional intervention strategies should fully account for the genetic background and physiological status of the target animals to develop tailored nutritional regimens.

Collectively, NCG promotes endogenous arginine synthesis, and activates the IGF-1/IRS2/PI3K/Akt/FoxO1 signaling axis and with coordinated alterations in purine and lipid metabolism. These molecular and compositional changes are consistent with, but do not establish, enhanced muscle growth or improved meat quality. This multi-omics study provides a mechanistic basis for the rational application of NCG in poultry production. These findings highlight that NCG, particularly at 800 mg/kg, serves as a promising precision feed additive for indigenous chicken breeds, with the dual benefit of enhancing muscle protein deposition and favorably altering nutrient and metabolic traits. These reversed transcriptomic patterns indicate that the highest tested level did not further improve the coordinated anabolic and purine-related responses.

This study measured mRNA expression only and did not assess protein abundance or phosphorylation of Akt and FoxO1; therefore, pathway activation remains inferred rather than demonstrated, and the mechanistic interpretation should be considered exploratory. Live body weight was recorded only on day 1 and day 35; although this design was adequate for calculating overall 1–35 d ADG, ADFI, and FCR, compensatory growth and phase-specific growth responses could not be assessed, and future studies should include intermediate weighing if growth trajectories are of interest. This study focused on growth performance, proximate composition, free amino acids, fatty acid composition, IMP, muscle histomorphology, transcriptomics, and metabolomics, but did not measure pH, meat color, water-holding capacity, drip loss, cooking loss, shear force, texture, lipid oxidation, protein oxidation, sensory attributes, or shelf-life; therefore, statements regarding eating quality, technological quality, oxidative stability, and consumer preference are not supported by the present data and should be regarded as hypotheses for future testing, and future studies should include these direct meat-quality measurements to determine whether the observed molecular and compositional changes translate into improved meat quality. In addition, although the actual NCG concentrations in the experimental diets were measured in the present batch and were close to target levels, batch-to-batch variation and possible losses during mixing, storage, or processing were not separately evaluated. No formal dose-optimization model was fitted; therefore, dose-related conclusions are restricted to the doses tested. Future studies should measure achieved NCG concentrations in independent batches and fit appropriate dose–response models to estimate the optimal dose and its confidence interval. Fatty acid data are reported as relative percentages of total identified FAMEs; no internal standard or response-factor correction was applied, and absolute quantification was not performed. Finally, only female Danzhou chickens were used in this study; therefore, the present findings may not be directly extrapolated to male birds, and sex-specific effects of NCG on muscle growth and metabolism remain to be investigated.

## 5. Conclusions

Dietary NCG at 400–800 mg/kg altered muscle composition, nutritional characteristics, and muscle development in Danzhou chickens; among the tested supplementation levels, 800 mg/kg produced the most favorable overall response for the measured traits. This effect was associated with altered mRNA expression of genes in the IGF-1/PI3K/Akt/FoxO1 signaling axis, including IRS2 upregulation and FOXO1 suppression at the transcript level. However, pathway activation was not directly demonstrated. Integrated multi-omics analyses suggested a possible association with purine metabolism, but causal roles and flavor effects require further validation. These findings outline a multilayered regulatory network through which NCG may modulate muscle growth, composition, and metabolism, providing a basis for precision nutritional interventions in indigenous chicken production. Direct meat-quality measurements are needed to determine whether these molecular and compositional changes improve eating quality, technological quality, oxidative stability, or consumer preference. These conclusions are limited to female Danzhou chickens; further studies including male birds are needed to determine whether the effects are sex-specific.

## Figures and Tables

**Table 2:** Effect of dietary NCG on the growth performance of Danzhou chickens.

Items	Treatments				SEM	*p*-Value		
	CON	N1	N2	N3		ANOVA	Linear	Quadratic
IBW, g	24.57	24.61	24.86	24.78	0.152	0.9060	0.5491	0.8421
FBW, g	182.97 ^b^	193.10 ^ab^	199.12 ^a^	190.31 ^ab^	2.002	0.0221	0.0722	0.0103
ADG, g	5.23 ^b^	5.52 ^ab^	5.69 ^a^	5.44 ^ab^	0.057	0.0221	0.0722	0.0103
ADFI, g	9.80	9.73	9.79	9.71	0.023	0.4197	0.3266	0.8584
FCR, g/g	1.88 ^a^	1.77 ^b^	1.72 ^b^	1.79 ^b^	0.018	0.0074	0.0224	0.0049

^a, b^ Means in the row without common superscripts are significant difference (*p* < 0.05). IBW, initial body weight; FBW, final body weight; ADG, average daily gain; ADFI, average daily feed intake; FCR, feed conversion ratio; CON (*n* = 6), basal diet; N1 (*n* = 6), basal diet plus 400 mg/kg NCG; N2 (*n* = 6), basal diet plus 800 mg/kg NCG; N3 (*n* = 6), basal diet plus 1200 mg/kg NCG.

**Table 3:** Effects of dietary NCG on the breast muscle composition of Danzhou chickens.

Items	Treatments				SEM	*p*-Value		
	CON	N1	N2	N3		ANOVA	Linear	Quadratic
Breast muscle								
Moisture, %	72.50	69.45	70.50	70.50	1.250	0.8718	0.6792	0.5693
Crude protein, %	19.94 ^c^	22.78 ^ab^	23.43 ^a^	20.61 ^b^^c^	0.502	0.0021	0.0005	0.0576
Fat, %	1.12	1.26	1.25	1.17	0.032	0.3431	0.5959	0.0890
Ash, %	1.07	1.05	1.09	1.08	0.038	0.9869	0.8362	0.9918
IMP (mg/g)	2.04	2.36	2.49	2.49	0.111	0.4517	0.1539	0.4710
Leg muscle								
Moisture, %	71.20	70.50	69.80	70.10	1.189	0.9817	0.7284	0.8459
Crude protein, %	21.10 ^b^	21.50 ^ab^	22.13 ^a^	20.89 ^b^	0.163	0.0242	0.9979	0.0077
Fat, %	1.15	1.20	1.18	1.22	0.036	0.9255	0.5822	0.9482
Ash, %	1.02	1.04	1.03	1.05	0.035	0.9936	0.8489	0.9913
IMP (mg/g)	2.10	2.33	2.36	2.19	0.063	0.4443	0.6157	0.1273

^a, b, c^ Means in the row with different superscripts indicate significant differences (*p* < 0.05). IMP, Inosine monophosphate; CON (*n* = 6), basal diet; N1 (*n* = 6), basal diet plus 400 mg/kg NCG; N2 (*n* = 6), basal diet plus 800 mg/kg NCG; N3 (*n* = 6), basal diet plus 1200 mg/kg NCG.

**Table 4:** Effects of dietary NCG on the free amino acid profile in breast muscle of Danzhou chickens.

Items mg/g	Treatments				SEM	*p*-Value		
	CON	N1	N2	N3		ANOVA	Linear	Quadratic
Aspartic acid	3.79	4.06	4.09	3.89	0.046	0.0530	0.3528	0.0096
Threonine	5.75	5.92	5.90	5.95	0.071	0.7845	0.3877	0.7036
Serine	15.85	15.65	15.25	15.25	0.341	0.9134	0.5032	0.8921
Glutamic acid	17.52	18.25	18.45	18.25	0.196	0.3684	0.1846	0.2448
Glycine	17.75	17.95	17.75	17.65	0.249	0.9823	0.8354	0.7829
Alanine	61.50	63.17	63.83	61.18	0.956	0.5893	0.7590	0.1895
Valine	6.15	6.05	6.22	6.25	0.069	0.7594	0.4673	0.6680
Isoleucine	4.18	4.40	4.35	4.25	0.044	0.2834	0.6846	0.0745
Tyrosine	6.15	6.17	6.15	6.08	0.035	0.8361	0.4978	0.5467
Phenylalanine	6.15 ^c^	6.37 ^ab^	6.45 ^a^	6.08 ^bc^	0.053	0.0259	0.7558	0.0037
Histidine	1.65	1.65	1.68	1.62	0.039	0.9669	0.8334	0.7115
Lysine	11.17	11.55	11.95	11.20	0.230	0.6236	0.8253	0.2443
Arginine	10.36 ^b^	11.80 ^a^	12.03 ^a^	11.80 ^a^	0.215	0.0127	0.0098	0.0286
Proline	15.80	15.58	15.46	15.35	0.349	0.9768	0.6635	0.9445
Cysteine	6.16 ^b^	6.40 ^a^	6.45 ^a^	6.27 ^ab^	0.041	0.0395	0.2750	0.0077
Methionine	1.83 ^b^	2.50 ^a^	2.67 ^a^	2.34 ^ab^	0.115	0.0488	0.0797	0.0240
Total	190.76	197.48	198.67	193.40	1.337	0.1254	0.4230	0.0260

^a, b, c^ Means in the row with different superscripts indicate significant differences (*p* < 0.05). CON (*n* = 6), basal diet; N1 (*n* = 6), basal diet plus 400 mg/kg NCG; N2 (*n* = 6), basal diet plus 800 mg/kg NCG; N3 (*n* = 6), basal diet plus 1200 mg/kg NCG.

**Table 5:** Effects of dietary NCG on the free amino acid profile in leg muscle of Danzhou chickens.

Items mg/g	Treatments				SEM	*p*-Value		
	CON	N1	N2	N3		ANOVA	Linear	Quadratic
Aspartic acid	3.41	3.61	3.63	3.50	0.079	0.7588	0.6949	0.3240
Threonine	5.18	5.33	5.33	5.37	0.078	0.8624	0.4644	0.7422
Serine	14.27	14.09	13.73	13.73	0.215	0.7804	0.3350	0.8429
Glutamic acid	15.77	16.43	16.61	16.44	0.221	0.5784	0.2941	0.3660
Glycine	15.98	16.16	15.98	15.89	0.401	0.9968	0.9079	0.8766
Alanine	55.35	56.86	57.45	55.06	0.932	0.7875	0.9747	0.3288
Valine	5.54	5.45	5.65	5.64	0.089	0.8617	0.5677	0.8428
Isoleucine	3.78	3.96	3.91	3.82	0.093	0.9056	0.9211	0.4878
Tyrosine	5.54	5.56	5.54	5.48	0.098	0.9938	0.8340	0.8513
Phenylalanine	5.54	5.74	5.81	5.48	0.103	0.6516	0.9087	0.2252
Histidine	1.49	1.49	1.52	1.46	0.044	0.9762	0.8877	0.7525
Lysine	10.05	10.39	10.75	10.08	0.236	0.7243	0.8395	0.3155
Arginine	8.99 ^b^	10.62 ^a^	10.82 ^a^	10.62 ^a^	0.253	0.0234	0.0155	0.0461
Proline	14.22	14.02	13.91	13.82	0.226	0.9420	0.5490	0.9100
Cysteine	5.51	5.76	5.1	5.40	0.126	0.8636	0.7247	0.4445
Methionine	1.96 ^b^	2.25 ^a^	2.21 ^a^	2.11 ^ab^	0.039	0.0305	0.1967	0.0091
Total	172.58	177.72	178.66	174.12	1.238	0.2610	0.6103	0.0582

^a, b^ Means in the row with different superscripts indicate significant differences (*p* < 0.05). CON (*n* = 6), basal diet; N1 (*n* = 6), basal diet plus 400 mg/kg NCG; N2 (*n* = 6), basal diet plus 800 mg/kg NCG; N3 (*n* = 6), basal diet plus 1200 mg/kg NCG.

**Table 6:** Effects of dietary NCG on the fatty acid composition in breast muscle of Danzhou chickens.

Items, %	Treatments				SEM	*p*-Value		
	CON	N1	N2	N3		ANOVA	Linear	Quadratic
Myristic (C14:0)	1.49	1.45	1.47	1.50	0.044	0.9847	0.9072	0.7444
Palmitic acid (C16:0)	28.32	27.66	27.73	27.90	0.366	0.9336	0.7365	0.6039
Margaric acid (C17:0)	0.23	0.24	0.24	0.25	0.021	0.9925	0.7704	0.9446
Stearic acid (C18:0)	13.89	12.92	13.11	13.74	0.466	0.8750	0.9497	0.4269
Arachidic acid (C20:0)	0.22	0.21	0.21	0.21	0.020	0.9991	0.8831	0.9947
Palmitoleic acid (C16:1)	3.98	4.02	4.06	4.11	0.152	0.9934	0.7738	0.9913
Elaidic acid (C18:1n9t)	0.15	0.16	0.21	0.17	0.01	0.1472	0.1220	0.2493
Oleic acid (C18:1n9c)	37.20	39.84	39.50	38.26	0.401	0.0663	0.3915	0.0141
Paullinic acid (C20:1)	0.24	0.27	0.28	0.25	0.012	0.7911	0.6913	0.3629
Linoleic acid (C18:2n6c)	10.27	9.98	9.95	9.92	0.183	0.9105	0.5341	0.7413
α-Linolenic acid (C18:3n3)	1.05	0.96	1.05	1.01	0.090	0.9848	0.9663	0.8783
Dihomo-γ-linolenic (C20:3n6)	0.43	0.36	0.39	0.42	0.023	0.7024	0.9926	0.2734
Arachidonic acid (C20:4n6)	1.90 ^a^	1.34 ^b^	1.25 ^b^	1.56 ^ab^	0.081	0.0117	0.0748	0.0038
Eicosapentaenoic (C20:5n3)	0.15	0.11	0.14	0.17	0.01	0.3464	0.3218	0.1847
Docosahexaenoic (C22:6n3)	0.48 ^ab^	0.47 ^bc^	0.41 ^c^	0.54 ^a^	0.013	0.0045	0.2482	0.0033
n-6 PUFAs	12.60	11.69	11.58	11.90	0.243	0.4700	0.3253	0.2232
n-3 PUFAs	1.68	1.54	1.60	1.71	0.093	0.9230	0.8560	0.5300
PUFAs n-6/PUFAs n-3	8.04	8.51	7.71	7.57	0.545	0.9410	0.6720	0.7940
∑SFA	42.66	41.04	41.29	42.09	0.447	0.5855	0.7290	0.2000
∑MUFA	41.57 ^b^	44.28 ^a^	44.05 ^a^	42.79 ^ab^	0.395	0.0430	0.2815	0.0097
∑PUFA	14.28	13.23	13.19	13.62	0.250	0.3973	0.3694	0.1527

^a, b, c^ Means in the row with different superscripts indicate significant differences (*p* < 0.05). ∑SFA, Total Saturated Fatty Acids; ∑MUFA, Total Monounsaturated Fatty Acids; ∑PUFA, Total Polyunsaturated Fatty Acids. n-6 PUFAs = C18:2n6c + C20:3n6 + C20:4n6; n-3 PUFAs = C18:3n3 + C20:5n3 + C22:6n3; CON (*n* = 6), basal diet; N1 (*n* = 6), basal diet plus 400 mg/kg NCG; N2 (*n* = 6), basal diet plus 800 mg/kg NCG; N3 (*n* = 6), basal diet plus 1200 mg/kg NCG. Values are expressed as the percentage of total identified FAMEs. Unidentified peaks were excluded. No internal standard was used; no response-factor correction was applied; absolute quantification was not performed. ΣSFA, total saturated fatty acids; ΣMUFA, total monounsaturated fatty acids; ΣPUFA, total polyunsaturated fatty acids.

**Table 7:** Effects of dietary NCG on the fatty acid composition in leg muscle of Danzhou chickens.

Items, %	Treatments				SEM	*p*-Value		
	CON	N1	N2	N3		ANOVA	Linear	Quadratic
Myristic (C14:0)	1.47	1.41	1.44	1.45	0.039	0.9693	0.9458	0.6937
Palmitic acid (C16:0)	28.27	26.96	27.08	27.05	0.359	0.5452	0.2868	0.3911
Margaric acid (C17:0)	0.23	0.23	0.25	0.24	0.010	0.9256	0.5847	0.8435
Stearic acid (C18:0)	13.79	12.69	12.81	13.37	0.275	0.4841	0.6518	0.1503
Arachidic acid (C20:0)	0.21	0.21	0.21	0.21	0.003	0.9940	0.8522	0.8452
Palmitoleic acid (C16:1)	4.13	4.14	4.20	4.22	0.093	0.9852	0.7101	0.9822
Elaidic acid (C18:1n9t)	0.17	0.17	0.17	0.18	0.004	0.3118	0.1871	0.4259
Oleic acid (C18:1n9c)	39.74	42.77	42.46	41.95	0.661	0.3782	0.2960	0.1927
Paullinic acid (C20:1)	0.26	0.28	0.26	0.26	0.003	0.1337	0.3232	0.0609
Linoleic acid (C18:2n6c)	9.07	8.79	8.77	8.66	0.154	0.8278	0.3969	0.7855
α-Linolenic acid (C18:3n3)	0.94	0.83	0.92	0.88	0.021	0.2817	0.7101	0.4089
Dihomo-γ-linolenic (C20:3n6)	0.23	0.21	0.23	0.23	0.005	0.5333	0.5414	0.2842
Arachidonic acid (C20:4n6)	1.05 ^a^	0.90 ^b^	0.85 ^b^	0.91 ^b^	0.023	0.0076	0.0120	0.0096
Eicosapentaenoic (C20:5n3)	0.12	0.11	0.12	0.12	0.002	0.0803	0.5242	0.0644
Docosahexaenoic (C22:6n3)	0.33 ^a^	0.29 ^ab^	0.25 ^b^	0.27 ^b^	0.009	0.0074	0.0034	0.0580
n-6 PUFAs	10.35	9.90	9.85	9.81	0.162	0.6376	0.2720	0.5422
n-3 PUFAs	1.38	1.23	1.29	1.27	0.024	0.1211	0.1700	0.1416
PUFAs n-6/PUFAs n-3	7.52	8.13	7.67	7.77	0.144	0.5190	0.8330	0.3950
∑SFA	43.97	41.52	41.79	42.32	0.537	0.3874	0.3387	0.1769
∑MUFA	44.29	47.36	47.08	46.61	0.636	0.3195	0.2480	0.1720
∑PUFA	11.73	11.12	11.13	11.07	0.175	0.5193	0.2279	0.4460

^a, b^ Means in the row with different superscripts indicate significant differences (*p* < 0.05). ∑SFA, Total Saturated Fatty Acids; ∑MUFA, Total Monounsaturated Fatty Acids; ∑PUFA, Total Polyunsaturated Fatty Acids. n-6 PUFAs = C18:2n6c + C20:3n6 + C20:4n6; n-3 PUFAs = C18:3n3 + C20:5n3 + C22:6n3; CON (*n* = 6), basal diet; N1 (*n* = 6), basal diet plus 400 mg/kg NCG; N2 (*n* = 6), basal diet plus 800 mg/kg NCG; N3 (*n* = 6), basal diet plus 1200 mg/kg NCG. Values are expressed as the percentage of total identified FAMEs. Unidentified peaks were excluded. No internal standard was used; no response-factor correction was applied; absolute quantification was not performed. ΣSFA, total saturated fatty acids; ΣMUFA, total monounsaturated fatty acids; ΣPUFA, total polyunsaturated fatty acids.

**Table 8:** Effects of dietary NCG on the breast muscle fiber traits of Danzhou chickens.

Items	Treatments				SEM	*p*-Value		
	CON	N1	N2	N3		ANOVA	Linear	Quadratic
Breast muscle								
Muscle fiber area (μm^2^)	477.24	579.75	574.05	507.20	18.967	0.1465	0.6036	0.0278
Muscle fiber Feret diameter (μm)	31.76	35.89	34.71	32.04	0.718	0.1047	0.9549	0.0184
Muscle fiber density (fibers/mm^2^)	1422.74 ^a^	1119.40 ^b^	1045.33 ^b^	1181.24 ^ab^	50.490	0.0360	0.0560	0.0210
Leg muscle								
Muscle fiber area (μm^2^)	475.00 ^b^	623.41 ^a^	636.89 ^a^	558.54 ^ab^	23.524	0.0464	0.1703	0.0129
Muscle fiber Feret diameter (μm)	32.75 ^b^	37.55 ^a^	37.83 ^a^	34.94 ^ab^	0.690	0.0168	0.1994	0.0033
Muscle fiber density (fibers/mm^2^)	1398.54 ^a^	1127.97 ^b^	1073.41 ^b^	1145.66 ^b^	44.823	0.0366	0.0305	0.0402

^a, b^ Means in the row with different superscripts indicate significant differences (*p* < 0.05). CON (*n* = 6), basal diet; N1 (*n* = 6), basal diet plus 400 mg/kg NCG; N2 (*n* = 6), basal diet plus 800 mg/kg NCG; N3 (*n* = 6), basal diet plus 1200 mg/kg NCG.

## Data Availability

The data presented in this study are openly available in NCBI at https://dataview.ncbi.nlm.nih.gov/object/PRJNA1452299 (accessed on 26 September 2026), reference number PRJNA1452299.

## Supplementary Materials

The following supporting information can be downloaded at https://www.mdpi.com/article/10.3390/foods15193516/s1. Table S1, Composition and nutrient components for basic diets, air-dried basis; Table S2, RNA-seq read depth and mapping statistics for each sample; Tables S3–S5, Spearman correlation coefficients and adjusted *p*-values for integrated transcriptome–metabolome correlation analysis (CON vs. N1, CON vs. N2, and CON vs. N3, respectively); Table S6, Adjusted *p*-values and gene counts for GO and KEGG enrichment terms; Table S7, Differential metabolites with FDR, −log10(P), VIP, and identification confidence; Figure S1, Transcriptome-associated bubble charts; Figure S2, Transcriptome-associated bubble charts; Figure S3, Correlation heatmaps of transcriptome–metabolome integrated analysis.

## Abbreviations

The following abbreviations are used in this manuscript:

*ABCA1*	*ATP binding cassette subfamily A member 1*
*ACTB*	*actin beta*
*ADF*	*acid detergent fiber*
*AKT1*	*AKT serine/threonine kinase 1*
CP	crude protein
*CPS-I*	*carbamyl phosphate synthetase I*
DAD	diode array detector
DEGs	differentially expressed genes
DHA	docosahexaenoic acid
DM	dry matter
FPKM	fragments per kilobase of transcript per million mapped reads
*FoxO1*	*forkhead box O1*
*GAPDH*	*glyceraldehyde-3-phosphate dehydrogenase*
GC-MS	gas chromatography–mass spectrometry
GO	Gene Ontology
HMDB	Human Metabolome Database
HPLC	high-performance liquid chromatography
*IGF1*	*insulin-like growth factor 1*
IMP	inosine monophosphate
*IRS2*	*insulin receptor substrate 2*
KEGG	Kyoto Encyclopedia of Genes and Genomes
ME	metabolizable energy
*MyoG*	*myogenin*
NCG	N-carbamylglutamate
NDF	neutral detergent fiber
OM	organic matter

## References

[1] Pan R., Qi L., Xu Z., Zhang D., Nie Q., Zhang X., Luo W. (2024). Weighted Single-Step GWAS Identified Candidate Genes Associated with Carcass Traits in a Chinese Yellow-Feathered Chicken Population. Poult. Sci..

[2] Philatha A., Rassmidatta K., Tanumtuen W., Ngoygudjik S., Blanch M., Viñado A., Wantanu S., Adeyemi K.D., Ruangpanit Y. (2026). Influence of Olive Pomace Extract on Performance, Gut Health, Immune Indices, Carcass Traits, and Meat Quality of Broiler Chickens Raised under Tropical Conditions. Poult. Sci..

[3] Wang Z., Zhu J., Ma Y., Liu T., Chen S., Gao M., Wang S., Wang J., Shen H. (2024). Effects of Isalo Scorpion Cytotoxic Peptide on Growth, Immune Performance, and Intestinal Flora of Yellow-Feathered Broilers. Front. Vet. Sci..

[4] Ai H., Lee Y.-Y., Lu Y., Tan C.P., Lai O.M., Li A., Zhang Y., Wang Y., Zhang Z. (2025). Effect of Structured Lipids as Dietary Supplements on the Fatty Acid Profile, Carcass Yield, Blood Chemistry, and Abdominal Fat Deposition of Female Broilers. Poult. Sci..

[5] Xie X., Shi L., Zhong Z., Wang Z., Pan D., Hou G., Xiao Q. (2024). Danzhou Chicken: A Unique Genetic Resource Revealed by Genome-Wide Resequencing Data. Poult. Sci..

[6] Peng W., Yang H., Cai K., Zhou L., Tan Z., Wu K. (2019). Molecular Identification of the Danzhou Chicken Breed in China Using DNA Barcoding. Mitochondrial DNA. B Resour..

[7] Abbas K., Raza A., Vasquez R.D., Roldan M.J.M., Malhotra N., Huang J.-C., Buenafe O.E.M., Chen K.H.-C., Liang S.-S., Hsiao C.-D. (2022). Ractopamine at the Center of Decades-Long Scientific and Legal Disputes: A Lesson on Benefits, Safety Issues, and Conflicts. Biomolecules.

[8] Liu Y., Liang S., Wang K., Zi X., Zhang R., Wang G., Kang J., Li Z., Dou T., Ge C. (2022). Physicochemical, Nutritional Properties and Metabolomics Analysis Fat Deposition Mechanism of Chahua Chicken No. 2 and Yao Chicken. Genes.

[9] Yang C., Wang W., Tang X., Huang R., Li F., Su W., Yin Y., Wen C., Liu J. (2022). Comparison of the Meat Quality and Fatty Acid Profile of Muscles in Finishing Xiangcun Black Pigs Fed Varied Dietary Energy Levels. Anim. Nutr..

[10] Yu B., Liu J., Zhang J., Mu T., Feng X., Ma R., Gu Y. (2022). Regulatory Role of RNA N6-Methyladenosine Modifications during Skeletal Muscle Development. Front. Cell Dev. Biol..

[11] Zhang Q., Mi M., Tianjiao E., Fu X., Bao N., Pan L., Zhao Y., Qin G. (2024). Effect of Synchronicity of Amino Acid Supply on the Synthesis of Protein in C2C12 Myotubes Cultured in Vitro. Front. Vet. Sci..

[12] Han Y., Kim D.H., Pack S.P. (2024). Marine-Derived Bioactive Ingredients in Functional Foods for Aging: Nutritional and Therapeutic Perspectives. Mar. Drugs.

[13] Yan X.-M., Zhang Z., Liu J.-B., Li N., Yang G.-W., Luo D., Zhang Y., Yuan B., Jiang H., Zhang J.-B. (2020). Genome-Wide Identification and Analysis of Long Noncoding RNAs in Longissimus Muscle Tissue from Kazakh Cattle and Xinjiang Brown Cattle. Anim. Biosci..

[14] Gu X., Lu S., Xu S., Li Y., Fan M., Lin G., Liu Y., Zhao Y., Zhao W., Liu X. (2025). Novel Oral Compound Z526 Mitigates Cancer-Associated Cachexia via Intervening NF-κB Signaling and Oxidative Stress. Genes Dis..

[15] Liu C., Ren N., Zhang H., Ma J. (2026). The Role of PYY in Improving Insulin Resistance. Front. Endocrinol..

[16] Wang T., Zhou D., Hong Z. (2025). Sarcopenia and Cachexia: Molecular Mechanisms and Therapeutic Interventions. MedComm.

[17] Zhang S., Xing Z., Ke J. (2025). Using β-Elemene to Reduce Stemness and Drug Resistance in Osteosarcoma: A Focus on the AKT/FOXO1 Signaling Pathway and Immune Modulation. J. Bone Oncol..

[18] Zhong Q., Liu Y., Correa M.R., Marconett C.N., Minoo P., Li C., Ann D.K., Zhou B., Borok Z. (2022). FOXO1 Couples KGF and PI-3K/AKT Signaling to NKX2.1-Regulated Differentiation of Alveolar Epithelial Cells. Cells.

[19] Zhao W., Li Z., Liu Q., Xie S., Li M., Wang Y., Li C. (2021). Analysis of Long Intergenic Non-Coding RNAs Transcriptomic Profiling in Skeletal Muscle Growth during Porcine Embryonic Development. Sci. Rep..

[20] Li M., Zhang N., Zhang W., Hei W., Cai C., Yang Y., Lu C., Gao P., Guo X., Cao G. (2021). Comprehensive Analysis of Differentially Expressed circRNAs and ceRNA Regulatory Network in Porcine Skeletal Muscle. BMC Genom..

[21] Liu Q., Akhtar M., Kong N., Zhang R., Liang Y., Gu Y., Yang D., Nafady A.A., Shi D., Ansari A.R. (2025). Early Fecal Microbiota Transplantation Continuously Improves Chicken Growth Performance by Inhibiting Age-Related Lactobacillus Decline in Jejunum. Microbiome.

[22] Zhang H., Zhao F., Peng A., Dong L., Wang M., Yu L., Loor J.J., Wang H. (2018). Effects of Dietary L-Arginine and N-Carbamylglutamate Supplementation on Intestinal Integrity, Immune Function, and Oxidative Status in Intrauterine-Growth-Retarded Suckling Lambs. J. Agric. Food Chem..

[23] Zhang Q., Zhang G., Zhang X., Yang J., Zhang Y. (2022). Effects of Inclusion of N-Carbamylglutamate in the Non-Protein Diet on Growth and Slaughter Performance, Meat Quality, Nitrogen Metabolism and Antioxidant of Holstein Bulls. Animals.

[24] Feng D., Yang Z., Li M. (2023). Dietary N-Carbamylglutamate Supplementation Improves Ammonia Tolerance of Juvenile Yellow Catfish Pelteobagrus Fulvidraco. Front. Physiol..

[25] Li Y.X., Moreira V.R., Wang L.S., Liu Q.L., Gao P.D., Zhang X.J., Fu X. (2023). N-Carbamylglutamate, a Promising Functional Feed Additive in Swine Production: A Review. Anim. Feed Sci. Technol..

[26] Zhao L., Tan S., Zhang W., Mao P., Wu X. (2026). N-Carbamylglutamate Improves Production Performance and Muscle Growth by Regulating Protein Digestive Function and Muscle Protein Synthesis in Broiler Chickens. Animals.

[27] Alderton W.K., Cooper C.E., Knowles R.G. (2001). Nitric Oxide Synthases: Structure, Function and Inhibition. Biochem. J..

[28] Wu G., Morris S.M. (1998). Arginine Metabolism: Nitric Oxide and Beyond. Biochem. J..

[29] Chacher B., Liu H., Wang D., Liu J. (2013). Potential Role of N-Carbamoyl Glutamate in Biosynthesis of Arginine and Its Significance in Production of Ruminant Animals. J. Anim. Sci. Biotechnol..

[30] Liu N., Zhang Z., Zhang J., Ma W., Wang C. (2025). Effects of N-Carbamylglutamate Supplementation on Cecal Morphology, Microbiota Composition, and Short-Chain Fatty Acids Contents of Broiler Breeder Roosters. Sci. Rep..

[31] Ma W., Dang D.X., Zhang J., Wang C., Li D. (2023). Effects of Dietary Supplementation of N-Carbamylglutamate on the Haematology Parameters, Secondary Sexual Characteristics and Testicular Gene Expression in Roosters. J. Anim. Physiol. Anim. Nutr..

[32] Wang Y.-H., Ayalew H., Wang J., Wu S., Qiu K., Qi G., Zhang H. (2024). N-Carbamylglutamate in Ovo Feeding Improves Carcass Yield, Muscle Fiber Development, and Meat Quality in Broiler Chickens. J. Sci. Food Agric..

[33] Wu H., Zhao D., Yu X., Chai H., Liu H., Peng W., Xu L., Hou G. (2026). N-Carbamylglutamate Promotes Growth and Immunity in Danzhou Chickens via Gut Microbiota-Metabolite Interactions Involving Sphingolipid and mTOR Pathways. Microorganisms.

[34] (2020). Formula Feeds for Replacement Pullets, Layers and Broilers.

[35] (2014). Determination of Moisture in Feedstuffs. General Administration of Quality Supervision.

[36] (2018). Determination of Crude Protein in Feedstuffs—Kjeldahl Method.

[37] (2025). Determination of Crude Ash in Feedstuffs.

[38] (2018). Determination of Calcium in Feedstuffs.

[39] (2018). Determination of Total Phosphorus in Feedstuffs—Spectrophotometric Method.

[40] (2022). Determination of Neutral Detergent Fiber (NDF) in Feedstuffs.

[41] (2022). Determination of Acid Detergent Lignin (ADL) in Feedstuffs.

[42] (2016). National Food Safety Standard—Determination of Moisture in Foods.

[43] (2016). National Food Safety Standard—Determination of Protein in Foods.

[44] (2016). National Food Safety Standard—Determination of Fat in Foods.

[45] (2016). National Food Safety Standard—Determination of Ash in Foods.

[46] Gao R., Liu H., Li Y., Liu H., Zhou Y., Yuan L. (2023). Correlation between Dominant Bacterial Community and Non-Volatile Organic Compounds during the Fermentation of Shrimp Sauces. Food Sci. Hum. Wellness.

[47] Figueiredo I.L., Claus T., Oliveira Santos Júnior O., Almeida V.C., Magon T., Visentainer J.V. (2016). Fast Derivatization of Fatty Acids in Different Meat Samples for Gas Chromatography Analysis. J. Chromatogr. A.

[48] Zhang X., Deng Y., Ma J., Hu S., Hu J., Hu B., Liu H., Li L., He H., Wang J. (2022). Effects of Different Breeds/Strains on Fatty Acid Composition and Lipid Metabolism-Related Genes Expression in Breast Muscle of Ducks. Poult. Sci..

[49] Arifin W.N., Zahiruddin W.M. (2017). Sample Size Calculation in Animal Studies Using Resource Equation Approach. Malays. J. Med. Sci..

[50] Yang J., Zheng J., Fang X., Jiang X., Sun Y., Zhang Y. (2021). Effects of Dietary N-Carbamylglutamate on Growth Performance, Apparent Digestibility, Nitrogen Metabolism and Plasma Metabolites of Fattening Holstein Bulls. Animals.

[51] Andersson U., Filipsson K., Abbott C.R., Woods A., Smith K., Bloom S.R., Carling D., Small C.J. (2004). AMP-Activated Protein Kinase Plays a Role in the Control of Food Intake. J. Biol. Chem..

[52] Rother K.I., Imai Y., Caruso M., Beguinot F., Formisano P., Accili D. (1998). Evidence That IRS-2 Phosphorylation Is Required for Insulin Action in Hepatocytes. J. Biol. Chem..

[53] Wang R., Li K., Sun L., Jiao H., Zhou Y., Li H., Wang X., Zhao J., Lin H. (2022). L-Arginine/Nitric Oxide Regulates Skeletal Muscle Development via Muscle Fibre-Specific Nitric Oxide/mTOR Pathway in Chickens. Anim. Nutr..

[54] He X., Xu J., Liu Y., Guo X., Wei W., Xing C., Zhang H., Wang H., Liu M., Jiang R. (2024). Explorations on Key Module and Hub Genes Affecting IMP Content of Chicken Pectoralis Major Muscle Based on WGCNA. Animals.

[55] Deng X., Ning Z., Li L., Cui Z., Du X., Amevor F.K., Tian Y., Shu G., Du X., Han X. (2023). High Expression of miR-22-3p in Chicken Hierarchical Follicles Promotes Granulosa Cell Proliferation, Steroidogenesis, and Lipid Metabolism via PTEN/PI3K/Akt/mTOR Signaling Pathway. Int. J. Biol. Macromol..

[56] Jiang M., Zhang K., Zhang Z., Zeng X., Huang Z., Qin P., Xie Z., Cai X., Ashrafizadeh M., Tian Y. (2025). PI3K/AKT/mTOR Axis in Cancer: From Pathogenesis to Treatment. MedComm.

[57] Gross D.N., van den Heuvel A.P.J., Birnbaum M.J. (2008). The Role of FoxO in the Regulation of Metabolism. Oncogene.

[58] Kitamura T., Ido Kitamura Y. (2007). Role of FoxO Proteins in Pancreatic Beta Cells. Endocr. J..

[59] Tran D.H., Kim D., Kesavan R., Brown H., Dey T., Soflaee M.H., Vu H.S., Tasdogan A., Guo J., Bezwada D. (2024). De Novo and Salvage Purine Synthesis Pathways across Tissues and Tumors. Cell.

[60] Villa E., Ali E.S., Sahu U., Ben-Sahra I. (2019). Cancer Cells Tune the Signaling Pathways to Empower de Novo Synthesis of Nucleotides. Cancers.

[61] Kondo M., Yamaoka T., Honda S., Miwa Y., Katashima R., Moritani M., Yoshimoto K., Hayashi Y., Itakura M. (2000). The Rate of Cell Growth Is Regulated by Purine Biosynthesis via ATP Production and G(1) to S Phase Transition. J. Biochem..

[62] Zhao W., Cai Z., Zhang J., Zhang X., Yu B., Fu X., Zhang T., Hu J., Shao Y., Gu Y. (2024). *PKM2* Promotes Myoblast Growth and Inosine Monophosphate-Specific Deposition in Jingyuan Chicken. Res. Vet. Sci..

[63] Remaley A.T., Stonik J.A., Demosky S.J., Neufeld E.B., Bocharov A.V., Vishnyakova T.G., Eggerman T.L., Patterson A.P., Duverger N.J., Santamarina-Fojo S. (2001). Apolipoprotein Specificity for Lipid Efflux by the Human ABCAI Transporter. Biochem. Biophys. Res. Commun..

[64] Remaley A.T., Thomas F., Stonik J.A., Demosky S.J., Bark S.E., Neufeld E.B., Bocharov A.V., Vishnyakova T.G., Patterson A.P., Eggerman T.L. (2003). Synthetic Amphipathic Helical Peptides Promote Lipid Efflux from Cells by an ABCA1-Dependent and an ABCA1-Independent Pathway. J. Lipid Res..

[65] Segrest J., Jones M., De Loof H., Brouillette C., Venkatachalapathi Y., Anantharamaiah G. (1992). The Amphipathic Helix in the Exchangeable Apolipoproteins: A Review of Secondary Structure and Function. J. Lipid Res..

[66] Fukunaga K., Imachi H., Lyu J., Dong T., Sato S., Ibata T., Kobayashi T., Yoshimoto T., Yonezaki K., Matsunaga T. (2018). IGF1 Suppresses Cholesterol Accumulation in the Liver of Growth Hormone-Deficient Mice via the Activation of ABCA1. Am. J. Physiol. Endocrinol. Metab..

[67] Cui D., Yu X., Guan Q., Shen Y., Liao J., Liu Y., Su Z. (2025). Cholesterol Metabolism: Molecular Mechanisms, Biological Functions, Diseases, and Therapeutic Targets. Mol. Biomed..

[68] Rytlewski G., Flis M., Jaworski H., Piórkowski J., Grela E.R. (2025). Effectiveness of black soldier fly (*Hermetia illucens*) as meal or whole larvae in feeding pheasants on production outcomes, chemical composition, and fatty acid profile of eggs (*Hermetia illucens*). Poult. Sci..

[69] Shu Y., Wu F., Yang W., Qi W., Li R., Zhang Z. (2024). Dietary Effect of Curcumin on Amino Acid, Fatty Acid, and Volatile Compound Profiles of Chicken Meat. Foods.

[70] Morris S.M. (2009). Recent Advances in Arginine Metabolism: Roles and Regulation of the Arginases. Br. J. Pharmacol..

[71] Wu G., Bazer F.W., Davis T.A., Kim S.W., Li P., Marc Rhoads J., Carey Satterfield M., Smith S.B., Spencer T.E., Yin Y. (2009). Arginine Metabolism and Nutrition in Growth, Health and Disease. Amino Acids.

[72] Pacher P., Beckman J.S., Liaudet L. (2007). Nitric Oxide and Peroxynitrite in Health and Disease. Physiol. Rev..

[73] Manning B.D., Toker A. (2017). AKT/PKB Signaling: Navigating the Network. Cell.

[74] Hu Y., Shao D., Wang Q., Xiao Y., Zhao X., Shen Y., Zhang S., Tong H., Shi S. (2019). Effects of Dietary N-Carbamylglutamate Supplementation on Growth Performance, Tissue Development and Blood Parameters of Yellow-Feather Broilers. Poult. Sci..

