# Supplementary material for: Dietary N-Carbamylglutamate Modulates Muscle Growth, Nutrient Composition, and Metabolic Profiles in Danzhou Chickens
Source: Foods. 2026 Oct 1;15(19):3516. doi: 10.3390/foods15193516 (PMC13665318; doi:10.3390/foods15193516)
Supplement: Supplementary file 1 [file foods-15-03516-s001.zip › foods-4580426-supplementary.pdf]

Table S1. Ingredient composition and nutrient levels of the basal diet on an air-dry basis.

Ingredients, %	Contents	Nutrient levels	Contents
Corn, %	63.30	ME[2], MJ/kg	11.98
Soybean meal (CP 44 %)	20.00	Dry matter[3], %	87.46
rapeseed meal	9.62	Crude protein[3], %	19.8
Soybean oil	1.40	Ash[3], %	5.44
Fish meal	1.50	Organic matter[3], %	94.56
Dicalcium phosphate	1.30	Lysine[2], %	1.14
Calcium carbonate	1.20	Tryptophan[2], %	0.21
Premix[1]	1.00	Valine[2], %	0.73
Salt	0.30	Methionine[2], %	0.39
L-Lys HCl (98.5%)	0.32	Methionine+ Cystine[2] %	0.74
DL-Met (99%)	0.06	Calcium[3], %	1.05
Total	100.00	Available phosphorus[2], %	0.47
		Neutral Detergent Fiber[3], %	10.60
		Acid Detergent Fiber[3], %	5.25

[1]Premix provides the following per kg of diet: vitamin A (retinyl acetate), 10,000 IU; vitamin D_3, 2500 IU; vitamin E, 20 mg; vitamin B_1, 1.5 mg; vitamin B_2, 3.5 mg; pantothenic acid, 10 mg; vitamin B_{12}, 0.01 mg; folic acid, 1 mg; niacin 30 mg; Choline chloride, 1000 mg; Cu ($CuSO_4 \cdot 5H_2O$), 8 mg; Fe ($FeSO_4 \cdot 7H_2O$), 80 mg; Zn ($ZnSO_4 \cdot 7H_2O$), 60 mg; Se ($NaSeO_3$), 0.15 mg; I (KI), 0.2 mg.

[2]Calculated value.

[3]Analysed content.

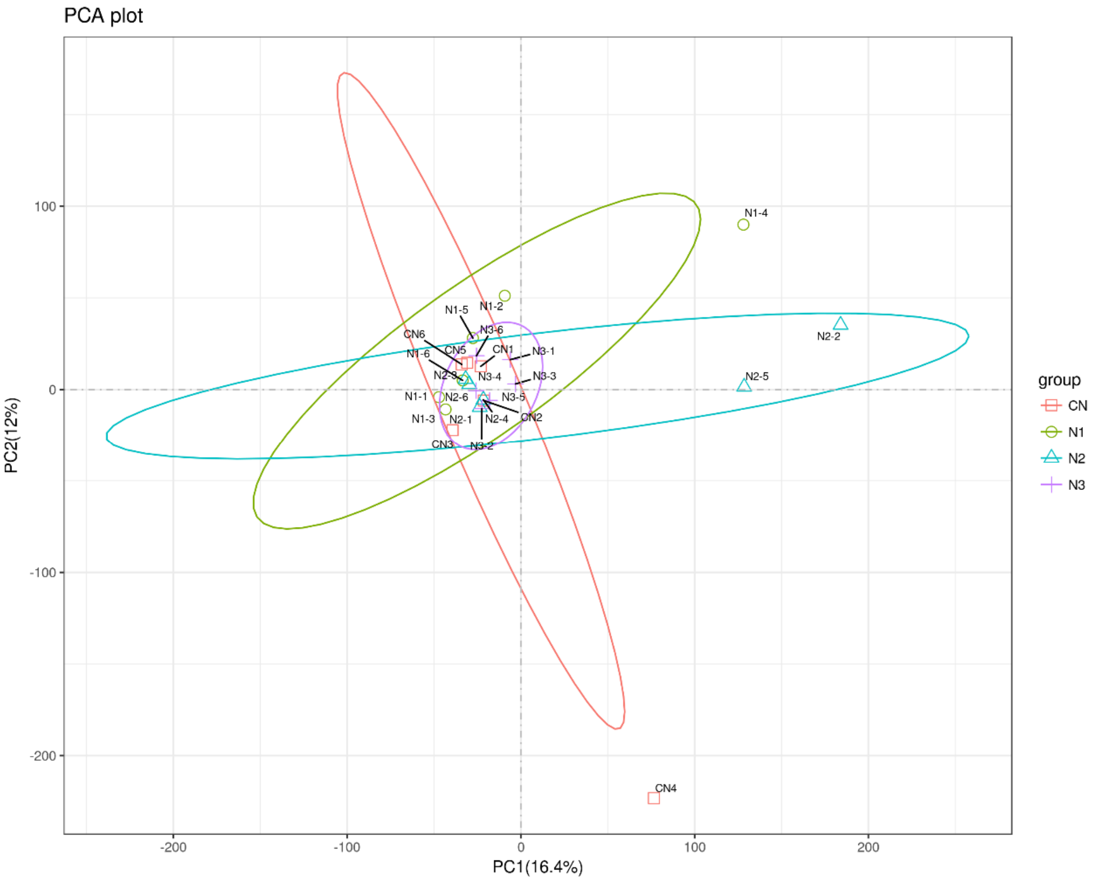

Figure S1. Transcriptome-associated bubble charts. A, Transcriptomics PCA plot. CON (n = 6), basal diet; N1 (n = 6), basal diet plus 400 mg/kg NCG; N2 (n = 6), basal diet plus 800 mg/kg NCG; N3 (n = 6), basal diet plus 1200 mg/kg NCG.

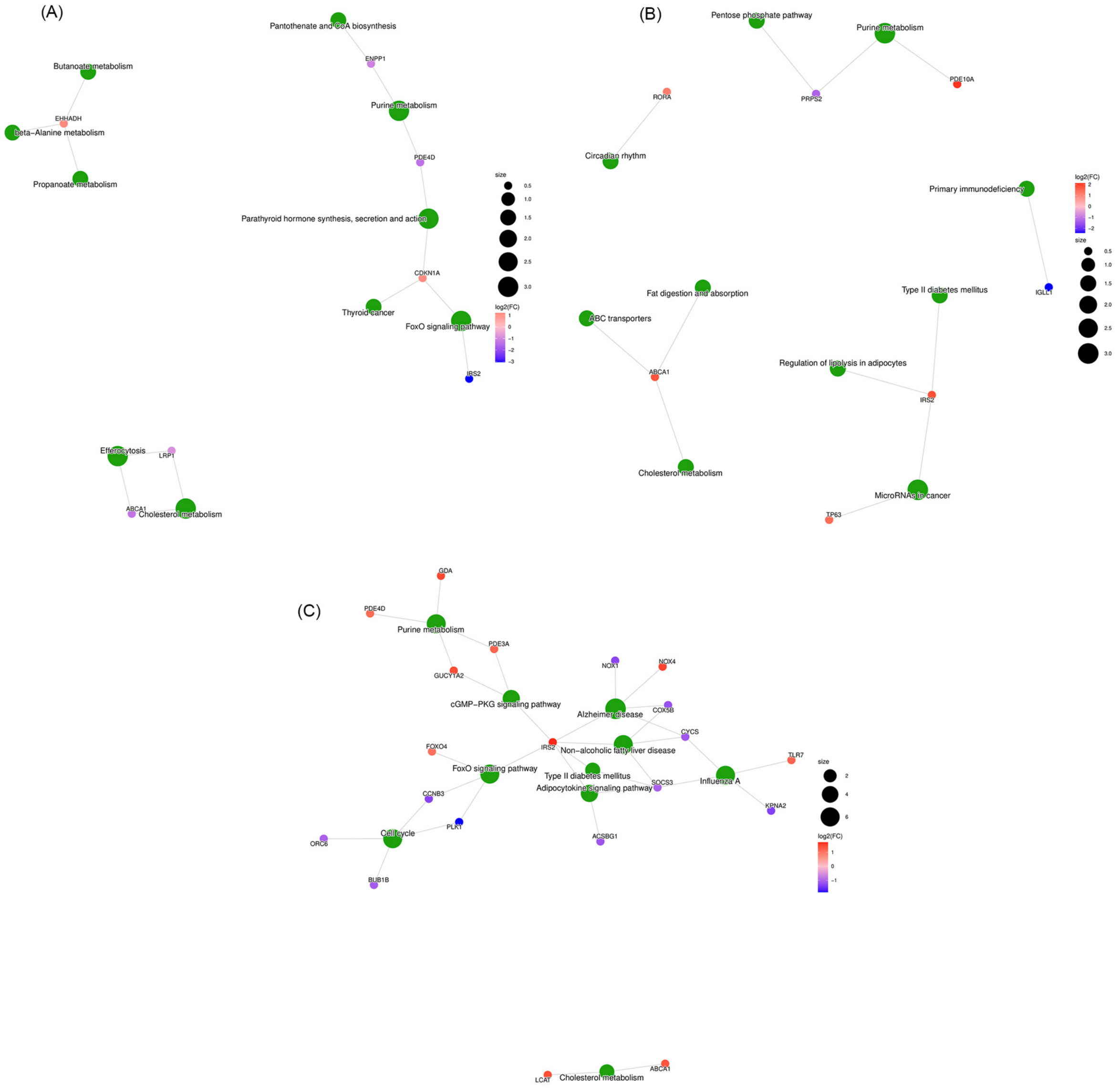

Figure S2. Transcriptome-associated bubble charts. A, Bubble chart of differentially expressed genes (DEGs) and their associated pathways between the CON and N1 group; B, Bubble chart of DEGs and their associated pathways between the CON and N2 group; C, Bubble chart of DEGs and their associated pathways between the CON and N3 group. CON (*n* = 6), basal diet; N1 (*n* = 6), basal diet plus 400 mg/kg NCG; N2 (*n* = 6), basal diet plus 800 mg/kg NCG; N3 (*n* = 6), basal diet plus 1200 mg/kg NCG.

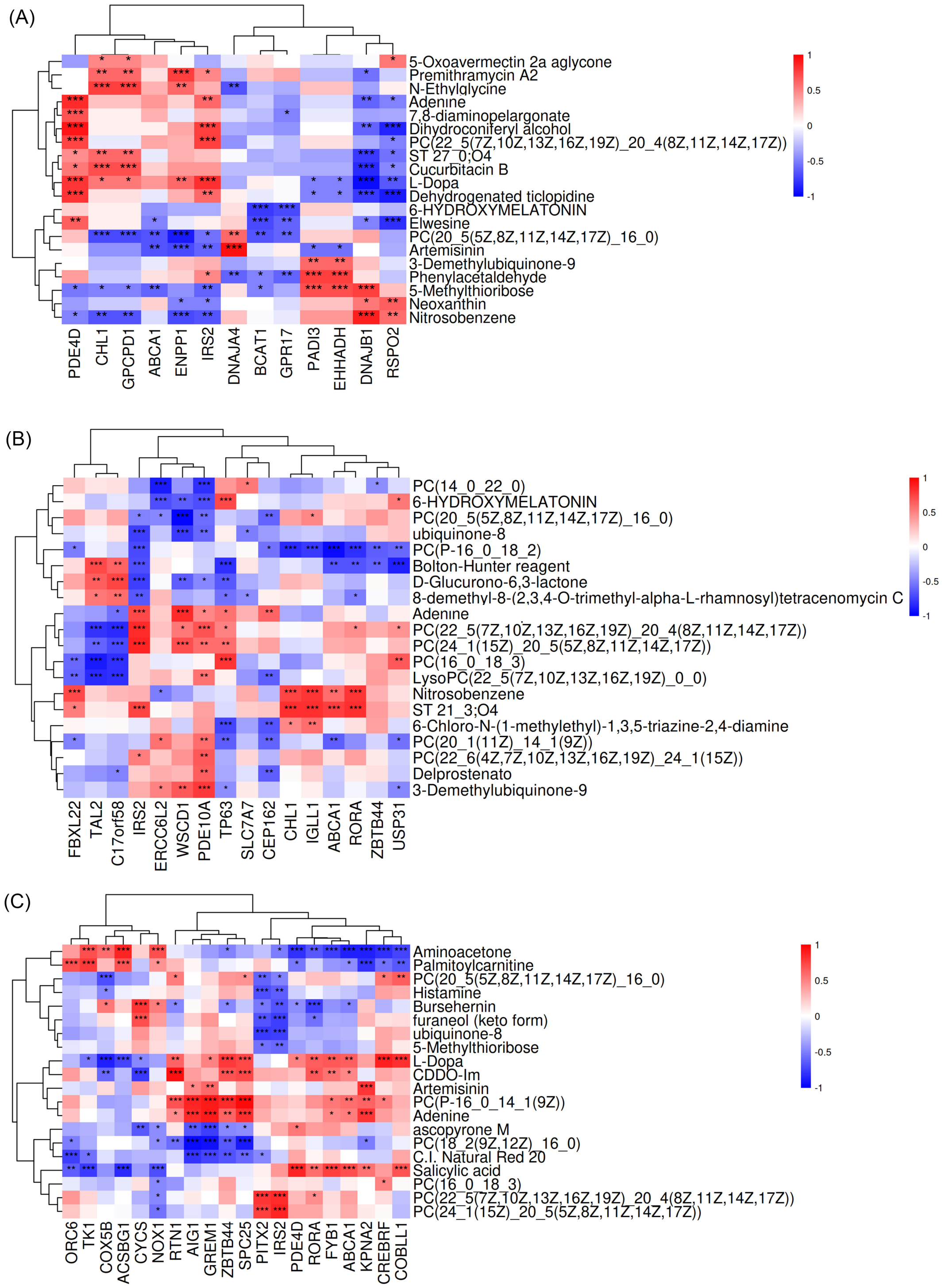

Figure S3. Correlation heatmaps between differentially expressed genes and differential metabolites. A, Correlation heatmap of DEGs between CON and N1; B, Correlation heatmap of DEGs between CON and N2; C, Correlation heatmap of DEGs between CON and N3. CON (n = 6), basal diet; N1 (n = 6), basal diet plus 400 mg/kg NCG; N2 (n = 6), basal diet plus 800 mg/kg NCG; N3 (n = 6), basal diet plus 1200 mg/kg NCG. * represents $P < 0.05$; ** represents $P < 0.01$; *** represents $P < 0.001$.
